# HypoMap—a unified single-cell gene expression atlas of the murine hypothalamus

**DOI:** 10.1038/s42255-022-00657-y

**Published:** 2022-10-20

**Authors:** Lukas Steuernagel, Brian Y. H. Lam, Paul Klemm, Georgina K. C. Dowsett, Corinna A. Bauder, John A. Tadross, Tamara Sotelo Hitschfeld, Almudena del Rio Martin, Weiyi Chen, Alain J. de Solis, Henning Fenselau, Peter Davidsen, Irene Cimino, Sara N. Kohnke, Debra Rimmington, Anthony P. Coll, Andreas Beyer, Giles S. H. Yeo, Jens C. Brüning

**Affiliations:** 1 Department of Neuronal Control of Metabolism, Max Planck Institute for Metabolism Research, Cologne, Germany; 2 Medical Research Council Metabolic Diseases Unit, Wellcome-MRC Institute of Metabolic Science - Metabolic Research Laboratories, University of Cambridge, Cambridge, UK; 3 Cambridge Genomics Laboratory, Cambridge University Hospitals NHS Foundation Trust, Cambridge, UK; 4 Department of Histopathology, Cambridge University Hospitals NHS Foundation Trust, Cambridge, UK; 5 Synaptic Transmission in Energy Homeostasis Group, Max Planck Institute for Metabolism Research, Cologne, Germany; 6 Cologne Excellence Cluster on Cellular Stress Responses in Aging-Associated Diseases (CECAD) and Center for Molecular Medicine Cologne (CMMC), University of Cologne, Cologne, Germany; 7 Center for Endocrinology, Diabetes and Preventive Medicine (CEDP), University Hospital Cologne, Cologne, Germany; 8 Novo Nordisk A/S, Måløv, Denmark; 9 Institute for Genetics, Faculty of Mathematics and Natural Sciences, University of Cologne, Cologne, Germany; 10 National Center for Diabetes Research (DZD), Neuherberg, Germany

**Keywords:** Hypothalamus, Computational biology and bioinformatics, Hypothalamus

## Abstract

The hypothalamus plays a key role in coordinating fundamental body functions. Despite recent progress in single-cell technologies, a unified catalog and molecular characterization of the heterogeneous cell types and, specifically, neuronal subtypes in this brain region are still lacking. Here, we present an integrated reference atlas, ‘HypoMap,’ of the murine hypothalamus, consisting of 384,925 cells, with the ability to incorporate new additional experiments. We validate HypoMap by comparing data collected from Smart-Seq+Fluidigm C1 and bulk RNA sequencing of selected neuronal cell types with different degrees of cellular heterogeneity. Finally, via HypoMap, we identify classes of neurons expressing glucagon-like peptide-1 receptor (*Glp1r*) and prepronociceptin (*Pnoc*), and validate them using single-molecule in situ hybridization. Collectively, HypoMap provides a unified framework for the systematic functional annotation of murine hypothalamic cell types, and it can serve as an important platform to unravel the functional organization of hypothalamic neurocircuits and to identify druggable targets for treating metabolic disorders.

## Abstract (web summary)

Steuernagel and Lam et al. present HypoMap, an integrated reference atlas of the murine hypothalamus based on 384,925 hypothalamic cells from publicly available single-cell sequencing datasets.

## Main

Hypothalamic neurocircuits are key regulators of integrative physiology and energy homeostasis^1,2^. In particular, the melanocortin neurocircuit, which comprises agouti-related peptide (AgRP)- and pro-opiomelanocortin (POMC)-expressing neurons in the hypothalamic arcuate nucleus (ARC), exerts effects on neurons in the hypothalamic paraventricular nucleus (PVH) and extra-hypothalamic projection sites, such as the bed nucleus of the stria terminalis (BNST)^3^, to control food intake and energy expenditure. Recently, studies have shown additional specialized neuronal subtypes located in the PVH and other hypothalamic regions, including the lateral (LH) and dorsomedial hypothalamus (DMH), that contribute to regulating energy homeostasis^4^. Single-cell RNA sequencing (sc-seq) experiments have revealed molecular heterogeneity of cell types that were previously considered homogeneous^5^, including POMC neurons^6^.

Many sc-seq datasets exist, covering multiple brain regions and conditions. However, direct comparison of these data is challenging owing to technical and experimental variations. The integration of datasets is a key step in projects such as the Human Cell Atlas^7^, the BRAIN Initiative, and the Cell Census Network (BICCN) (https://biccn.org/data). Recently, BICCN released an integrated single-cell reference for the primary motor cortex across different data modalities and species, underscoring the power that the analysis of systematically collected data on brain cell types and their connections can provide^8^. The emergence of dedicated portals and applications, such as Azimuth (https://azimuth.hubmapconsortium.org/), to facilitate access to reference datasets, further highlights the usefulness of combining available resources with newly generated data^9^.

The field of sc-seq data integration is evolving rapidly, with more than 20 available algorithms^10^. These methods use different approaches, such as shared low-dimensional embeddings (Seurat)^11^, soft-clustering strategies (Harmony)^12^, identification of nearest neighbors across datasets (Scanorama, fastMNN)^13,14^, and deep-learning strategies like variational auto-encoders (scVI)^15^. Additionally, traditional approaches developed for bulk RNA-seq, such as Combat^16^, are widely used. The two key aims of these methods are: (1) to mix datasets and correct for the technical differences originating from experimental variation, while (2) retaining the underlying biological information in each cell type.

Additionally, single-nucleus sequencing (nucSeq) has gained a lot of attention in recent years. The major advantage of nucSeq is removal of the time-consuming enzymatic cell-dissociation steps, which can potentially influence gene expression^17^. In addition, nucSeq can be performed on frozen tissues, thus simplifying logistics, especially when dealing with precious human materials. Recent studies have shown that nucSeq is largely comparable to sc-seq, despite profiling different RNA species^18,19^.

Here, we attempted to create the first murine hypothalamic reference, ‘HypoMap,’ by systematically evaluating different integration algorithms to choose the best approach for integrating data from 17 published datasets and an in-house hypothalamic nucSeq dataset from ad-libitum-fed and overnight-fasted mice. We validated HypoMap by comparing the transcriptomic profiles to: (1) sc-seq data collected from traditional Smart-Seq+Fluidigm C1 (ref. ^20^); and (2) selected cell populations through bulk bacterial artificial chromosome-translating ribosome affinity purification (bacTRAP) RNA-seq.

To further demonstrate the use of HypoMap, we molecularly and spatially characterized neurons expressing glucagon-like peptide-1 receptor (*Glp1r*) and prepronociceptin (*Pnoc*), identified from HypoMap and bacTRAP. GLP-1 is an incretin hormone secreted from the gut that has an important role in the control of food intake and satiety^21,22^. GLP-1R agonists (GLP-1RA) are used clinically to treat type 2 diabetes and obesity, with recent studies showing that they exert their effects in the area postrema (AP)^23^; however, their effects in the hypothalamus are less known^24,25^. Similarly, we have recently identified PNOC neurons in the ARC (PNOC^ARC^) as a GABAergic cell population, which is readily activated upon consumption of calorie dense, highly palatable food. Activation of PNOC^ARC^ neurons promotes food intake; conversely, the ablation of these neurons prevents high-fat-diet-induced hyperphagia and weight gain^26^. Thus, PNOC^ARC^ neurons represent a promising target for the treatment of obesity. Yet, a clear molecular definition of these neurons is still lacking.

## Results

### The generation of HypoMap

To develop a unified hypothalamic cell atlas comprising cell types from major hypothalamic regions, we combined 17 publicly available droplet-based hypothalamus sc-seq datasets^5,27–41^ covering different hypothalamic regions, from the preoptic area (POA) to the ventroposterior hypothalamus (VPH) (Supplementary Table 1). In addition, we performed nucSeq of 36,626 nuclei isolated from hypothalami of mice that had been either ad libitum chow fed or fasted overnight. This brought the total number of hypothalamic cells/nuclei, after quality control, to 384,925 (Supplementary Table 1).

**Fig. 1:**
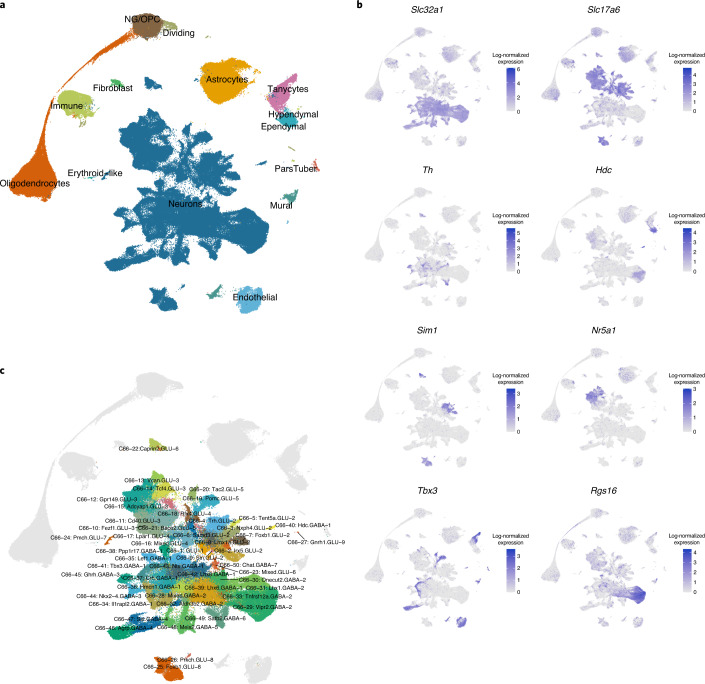
Unified hypothalamus reference map. Integration of 17 single-cell sequencing datasets into one harmonized reference. **a**, UMAP visualization of HypoMap, colored by major cell types. **b**, UMAP of neuronal clusters in HypoMap (other cell types in gray) **c**, UMAP expression of key neuronal type markers and regional markers in each cell. Color corresponds to log-normalized expression values scaled to the maximum of each gene. Source data

**Extended Data Fig. 1:**
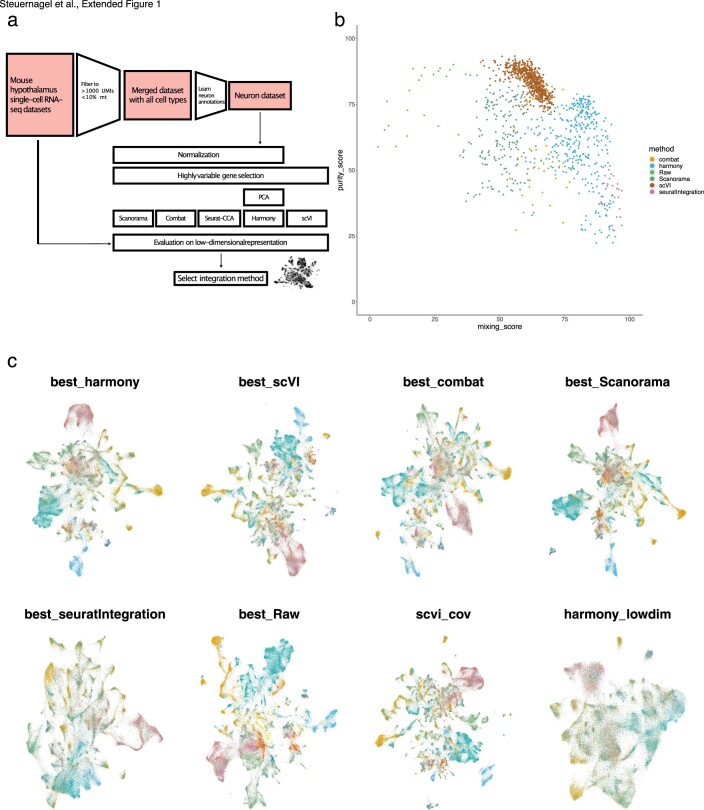
Selection of single-cell integration method. **a**, Overview of the pipeline used to determine an optimal integration of data including normalization and feature selection. **b**, Evaluation metrics calculated on integration results of preliminary dataset (85,000 cells). Purity refers to a combined score of cell type purity and cluster separation. Mixing refers to a combined score of dataset mixing. An optimal integration achieves high mixing scores while retaining high purity scores. **c**, UMAPs of each integration method’s best result colored by dataset of origin to show dataset mixing and cluster separation. As indicated by the metrics, most methods are able to mix the data and retain most of the original cell types. The *Raw* PCA is not mixing the data fully, while the low-dimensional Harmony result is not able to represent the full complexity of the data. Source data

**Extended Data Fig. 2:**
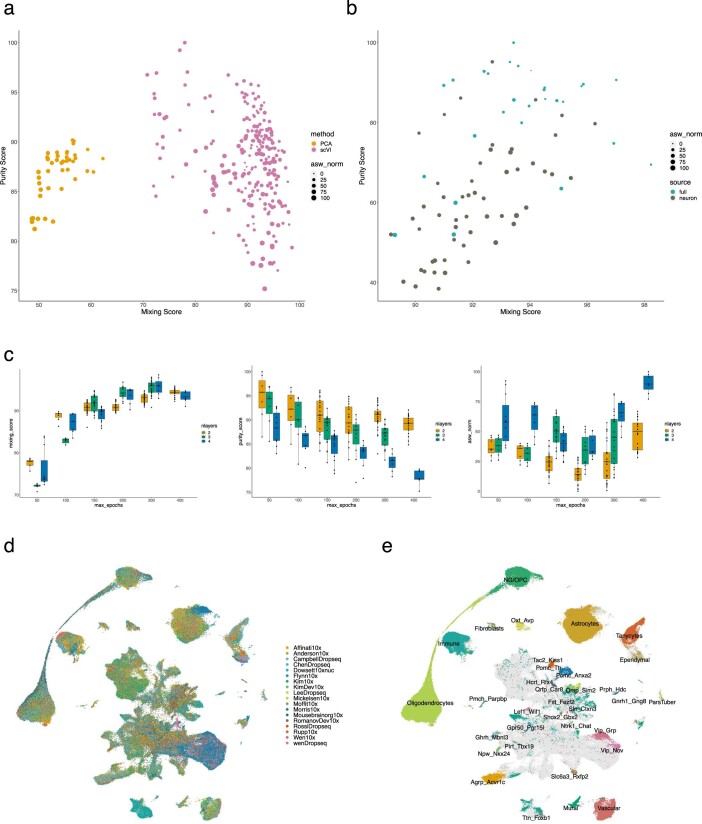
Optimization of scVI parameters. **a**, Evaluation metrics calculated on scVI integration results of full HypoMap (384,925 cells). Purity refers to cell type purity only. Cell type separation (asw_norm = average silhouette width) is shown by the point size (see methods for details on metrics). PCA (orange) clearly mixes that data less well than scVI (pink). **b**, Evaluation metrics similar to (**a**), calculated on scVI integration results with comparable hyperparameters using either all cells (light blue) or only neurons (grey) as input. Using all cells as input did not affect the integration performance in mixing and purity, but the the cluster separation (asw_norm) was lower. **c**, Example box plots for detailed evaluation of scVI hyperparameters, visualizing the influence of the number of training rounds (epochs) and hidden layers on the three different metrics. Each point corresponds to a scVI training run on the full HypoMap data. The center of the boxplot is the median of all runs, the lower and upper hinges correspond to the first and third quartiles and the whiskers extend from each hinge to the largest value smaller than 1.5 times the distance between the first and third quartiles. Overall n = 224 scVI runs that were compared, the number differs between boxplots depending on the parameters. **d**, UMAP visualization of HypoMap colored by datasets to visualize mixing. **e**, UMAP visualization of HypoMap colored by mapped cell types for the evaluation of purity (see Methods and Supplementary Table 2 for details). Source data

Next, we systematically evaluated the data-integration algorithms Harmony, Scanorama, scVI, Combat and Seurat (CCA) across different parameter ranges, and benchmarked their performance on the basis of batch mixing, cell-type purity, and cluster separation (see Supplementary Information, Extended Data Fig. 1, and Supplementary Table 2 for details). We found that scVI consistently achieved the highest cell-type purity scores while retaining high cluster separation and good dataset mixing. Other methods, such as Seurat (CCA), achieved higher mixing scores, but performed worse in retaining cell-type purity (Extended Data Fig. 1b). Therefore, we proceeded with scVI and further optimized the hyperparameters on the combined dataset (see Methods and Extended Data Fig. 2) to generate the final integrated reference dataset—HypoMap, visualized here via uniform manifold approximation and projection (UMAP) in Figure 1a.

The majority of cells in HypoMap are neurons (56.9%, blue) (Fig. 1a,c), followed by astrocytes (13%, golden brown), oligodendrocytes (12.7%, orange), and oligodendrocyte precursor cells (OPCs, 9.5%, brown). HypoMap can distinguish rarer cell types, such as microglia (3.7%, light-green), endothelial cells (2.6%, light blue), tanycytes (2.5%, pink), ependymal cells (1.1%, cyan), and mural cells (0.9%, turquoise) (Fig. 1a). Figure 1b shows the expression of key neuronal markers: VGlut2 (*Slc17a6*), VGat (*Slc32a1*), *Th*, and *Hdc*. We also examined the expression of regional markers, such as *Sim1* for PVH, *Nr5a1* (Sf-1) for ventromedial hypothalamus (VMH), *Tbx3* for ARC, and *Rgs16* for suprachiasmatic nucleus (SCN), which highlight the spatial origin of the neurons as one of major driving factors for segregation.

**Extended Data Fig. 3:**
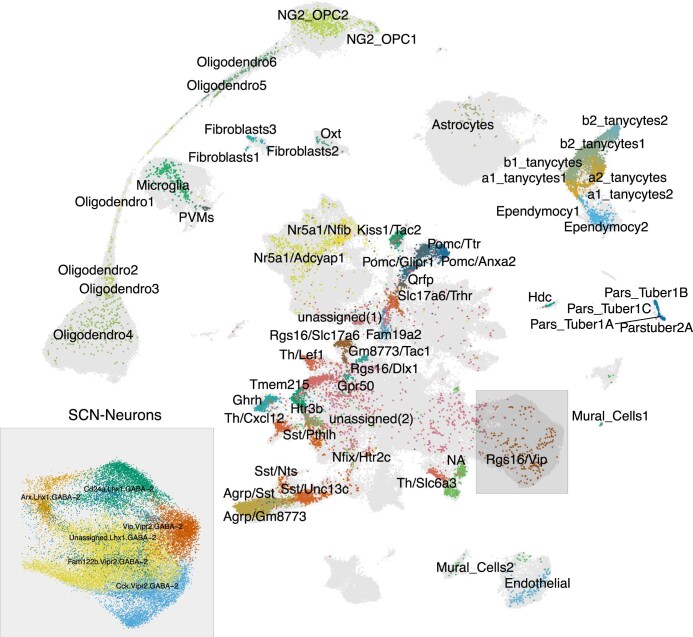
UMAP visualization of cells and original annotations from Campbell et al. UMAP visualization of cells and original annotations from Campbell et al., highlighting which parts of HypoMap are covered by this dataset. Inset shows the enlarged view of cell clusters from the SCN colored by HypoMap clusters. Source data

Each of the 18 datasets is distributed across multiple cell types in HypoMap (Extended Data Fig. 2d), with some areas showing over- or under-representation of cells from specific datasets. This is expected owing to the anatomically restricted sampling strategies used in some of these studies (for example Morris et al.^40^, SCN in deep blue; Kim et al.^32^, VMH in dark green) (Extended Data Fig. 2d). We examined the author annotations of the ARC cells from Campbell et al.^5^ more specifically, and the dataset covers only a subset of HypoMap, as expected (Extended Data Fig. 3). Cell types identified in this study largely consist of populations from the ARC, but we also observed cell types from other regions, for example the VMH (Extended Data Fig. 3) and the pituitary, as discussed in the original study^5^.

**Fig. 2:**
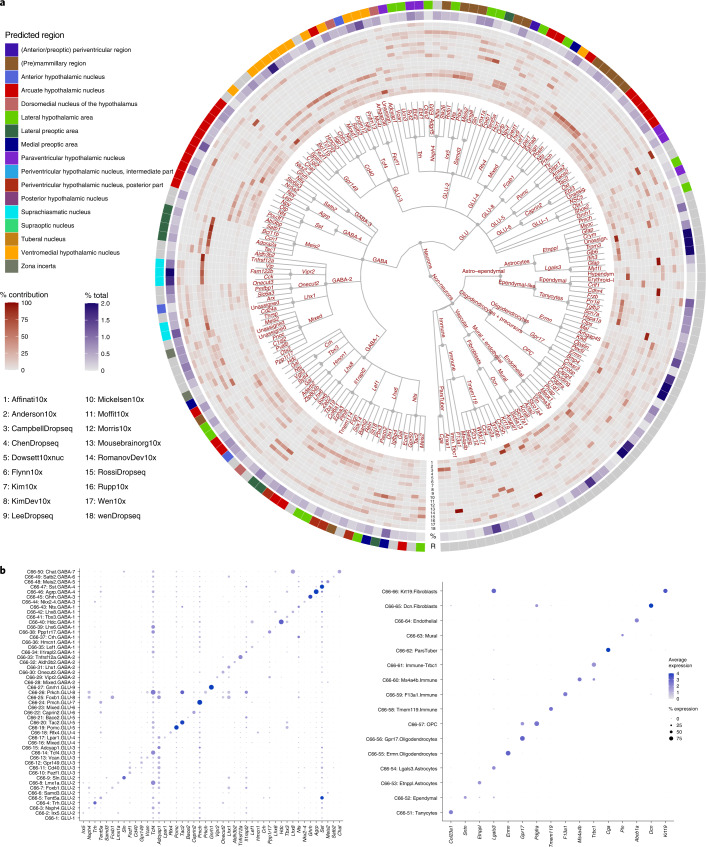
Harmonized annotation of hypothalamus cell types. **a**, A circular hierarchical tree of clusters of HypoMap. The first 5 levels with up to 185 clusters are shown, highlighting the diversity of hypothalamic cells when combining data across regions. Individual clusters at levels 4 and 5 are named with the most informative marker gene, given as edge labels. The inner (red) circular heatmap depicts the percentage contribution of each dataset to the clusters at the lowest tree level. The middle heatmap (blue) depicts the relative percentage contribution of each cluster at the lowest tree level to the total cell number. The scale is limited to 2%. The outer ring depicts the most likely region of origin (R) for each neuron cluster on the lowest level of the displayed tree. If support was insufficient for a cluster, no region was assigned, and the cluster was colored gray (see Methods). **b**,**c**, Dot plots displaying marker genes used for annotating the clusters at level 4 (C66) of the tree in **a**. For clusters with a proper name (for example, ‘Astrocytes’), the most specific gene that would have been used for annotation is included. Dot color corresponds to average log-normalized expression levels of each gene in a cluster and dot size to the percentage of cells expressing a marker in the cluster. **b**, Neuronal cell types. **c**, Non-neuronal cell types. See also Supplementary Tables 5 and 6. Source data

**Extended Data Fig. 4:**
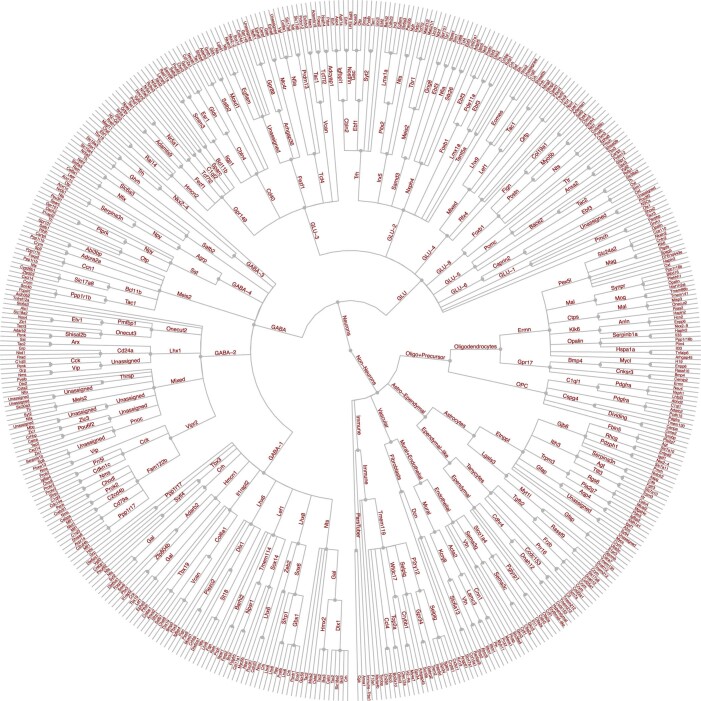
Full hierarchical cluster tree. *Full hierarchical cluster tree* of HypoMap showing all 7 cluster levels. This includes level 6 (C286) and 7 (C465). Individual clusters at levels 4 – 7 are named with the most informative marker gene, given as edge labels. Full cluster names were constructed by concatenating the given gene names with those of all ancestors. Source data

**Extended Data Fig. 5:**
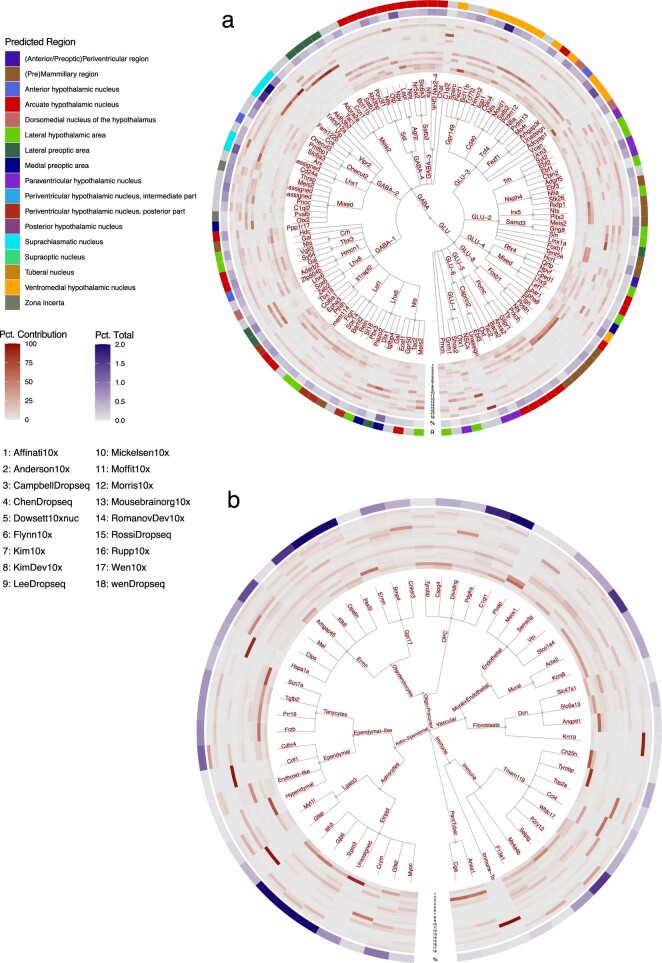
Hierarchical cluster trees of HypoMap split into neuronal (A) and non-neuronal (B) populations. Similar to Fig. 2A, but split into neuronal (a) and non-neuronal (b) populations. The first 5 levels with up to 185 clusters are shown. Individual clusters at levels 4 and 5 are named with the most informative marker gene, given as edge labels. The inner (red) circular heatmap depicts the contribution of each dataset to the clusters at the lowest tree level in percent. The middle heatmap (blue) depicts the relative contribution of each cluster at the lowest tree level to the total cell number in percent. The scale is limited to 2%. The outer ring depicts the most likely region of origin (R) for each neuron cluster on the lowest level of the displayed tree. If support was insufficient for a cluster, no region was assigned and the cluster was colored in grey (see methods). For the non-neuronal cell types in (b) no regional prediction was conducted. Source data

To construct a unified set of cell annotations, we adopted a multi-level clustering of cell populations using the Leiden algorithm^42^ and Multiresolution Reconciled Tree (mrtree)^43^ (see Methods). This resulted in a circular dendrogram (or tree) representing the underlying hierarchical organization of cell populations, similar to atlases of the brain transcriptome published previously^20,29^. Tree pruning was achieved by merging clusters that could not be separated by marker genes. The final clusters represent an overview of the transcriptomic landscape of the ‘sequenced’ hypothalamus (Fig. 2a and Supplementary Table 3). In total, we generated seven levels of clusters, each with increasing granularity, although here we show only the top 5 levels, C2, C7, C25, C66, and C185 (Fig. 2a); the two lowest levels (C286 and C465) are hidden to retain visual clarity (Supplementary Table 4; the full tree and a split version of Fig. 2a are shown in Extended Data Figs. 4 and 5, respectively).

**Extended Data Fig. 6:**
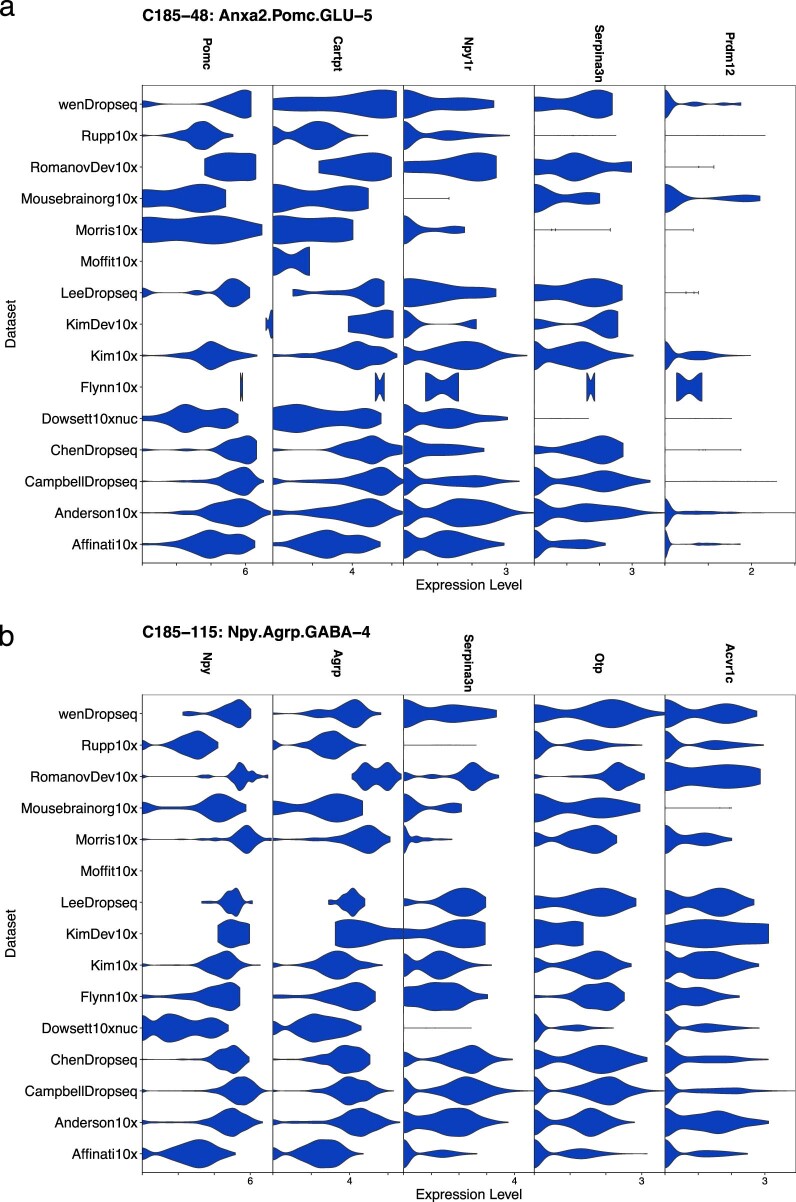
Marker gene expression across datasets. **a-b**, Violin plots showing the expression of the top 5 marker genes (selected by specificity and adjusted p-value) of POMC subcluster C185-48: Anxa2.Pomc.Glu-5 (a) and AgRP subcluster C185-115: Npy.Agrp.GABA-4 across datasets, demonstrating that the expression level of key marker genes are mostly stable. Source data

We next carried out differential gene expression (DEG) analysis to determine marker genes for all nodes at all cluster levels (Supplementary Tables 5 and 6). Each node of the tree was labeled using the most informative marker gene (see Methods). The full cluster annotation was constructed by concatenating the labels from all ancestor node(s), thereby incorporating the hierarchical structure (Extended Data Fig. 6 shows the marker expression of AgRP and POMC clusters across different source datasets). Additionally, for the highest three cluster levels, we manually annotated well-described cell type labels where applicable. Figure 2b,c shows dot plots of marker genes used for annotating the clusters at level C66 for neurons and non-neuronal populations, respectively.

As shown in Figure 2a, the top level of the tree separates cells into neurons and non-neuronal populations; this is followed by seven clusters at the second level (C7), which further segregate cells into excitatory glutamatergic (GLU) and inhibitory GABAergic neurons (GABA) (See also Fig. 1c): glial cells, including astrocytes and ependymal cells (*Gja1*); oligodendrocytes and precursor cells (*Sox10*); microglia (*Ly86*); vascular cell types, including fibroblasts, mural cells, and endothelial cells (*Igfbp7*); and pars tuberalis cells (*Cga*) (Fig. 2a).

### Neuronal populations

Focusing on the subset of 219,030 neurons, the next cluster levels consist of 16 (C-25) and 50 (C-66) clusters that further subdivide the GLU and GABA subtrees. An example of a well-defined cluster is the Pomc.GLU-54 neuronal cluster, which includes three subclusters: Anxa2.Pomc.GLU-5, Ttr.Pomc.GLU-5, and Glipr1.Pomc.GLU-5, consistent with Campbell et al.^5^. The lowest level depicted in the tree (C185) consists of 130 neuronal cell types (Fig. 2a). Interestingly, by combining all datasets together, we were able to identify a cluster of 61 extremely rare *Gnrh1*-expressing neurons (Gnrh1.GLU-9) (Fig. 2a).

The inner heatmap ring in Figure 2a depicts the contribution of each dataset. As expected, datasets that cover specific regions contribute strongly to clusters originating from that region, but little to other clusters (for example, Wen et al.^31^); other, less selective, datasets, such as Chen et al.^34^, and the in-house nucSeq cover a larger subset of the tree. Crucially, we found that no single dataset contributes to all clusters, thus emphasizing the power of the harmonized clustering on the basis of the integrated data of HypoMap. The middle ring in the heatmap in Figure 2a shows how each cluster contributes to the total number of cells in HypoMap (also in Supplementary Table 7).

Next, we performed spatial predictions for neuronal clusters, employing per-voxel enrichment analysis by overlapping the in situ hybridization data from the Allen Brain Atlas (https://brain-map.org) and cluster gene markers, followed by manual curation using known spatial origins of source datasets (see Methods). The predicted region annotation is shown in the outer ring (Fig. 2a and Supplementary Table 8). We found that regions with well-defined gene markers, such as the ARC, VMH, and SCN, were annotated with high confidence, consistent with annotations from the original studies. We also identified clusters originating from LH, such as Hcrt.Rfx4.GLU-4, which co-expresses *Hcrt* and *Pydn*, and *Pmch* neurons (Pmch.GLU-7), consistent with Rossi et al.^35^ and Mickelsen et al.^33^. The DMH is a region that lacks distinctive gene marker(s); at C185, three clusters were predicted to originate from the DMH (two glutamatergic and one GABAergic clusters, Fig. 2a).

**Fig. 3:**
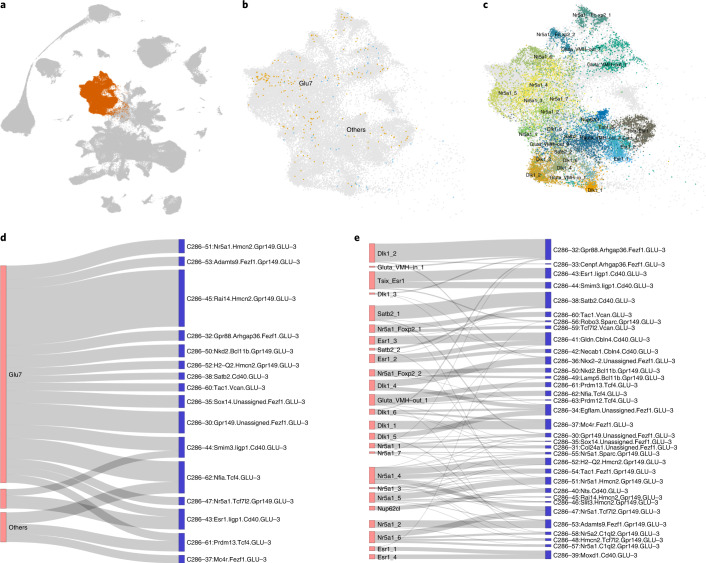
Comparison of HypoMap and original clusters. **a**, HypoMap UMAP highlighting the cluster C25-3: GLU-3, which contains *Nr5a1*- and *Fezf1-*expressing neuronal populations from the VMH that are compared in **b**–**e**. **b**,**c**, UMAP plot of cells from the C25-3: GLU-3 cluster from Chen et al.^34^ (**b**) and Kim et al.^32^ (**c**) overlayed on all cells of the cluster (gray) and colored by the original author annotations. **d**,**e**, Sankey diagrams showing the original author annotations of Chen et al. (**d**) and Kim et al. (**e**), compared with the HypoMap subclusters (C286) of C25-3: GLU-3. Chen et al. (**d**) covered VMH neurons only sparsely, and the combination with other datasets greatly improves cell classification. The VMH-specific dataset from Kim et al. (**e**) covered most subclusters identified in HypoMap, although in some cases clusters were further partitioned. (See Supplementary Table 20 for a full overview of all original and HypoMap cell labels). Source data

The distribution of ARC, VMH, and SCN neurons over multiple HypoMap clusters indicates that the larger cell numbers from additional datasets enhance the clustering granularity, thus allowing for more accurate stratification of cellular subtypes. For example, we observed a refinement of the clustering of VMH *Nr5a1*- and *Fezf1*-expressing populations (C25-3: GLU-3) in HypoMap (Fig. 3a), compared with the original annotations from Chen et al.^34^ (Fig. 3b,d); this is largely due to the integration with VMH-specific datasets, such as from Kim et al.^32^, while retaining clustering granularity, even when compared with the original annotations from Kim et al.^32^. (Fig. 3c,e). The improved clustering sensitivity also allowed the assignment of previously unlabeled cells in Campbell et al.^5^ into more distinct clusters, such as *Cck*-expressing Cck.Vipr2.GABA-2 cells (C185-73), which were previously annotated simply as Rgs16/Vip (Extended Data Fig. 3 inset).

### Non-neuronal populations

There are 165,895 non-neuronal cells in HypoMap. Non-neuronal cells exhibit a lower level of heterogeneity, despite being sampled from different hypothalamic regions, and this is reflected in fewer branches in the tree (Fig. 2a).

The majority of non-neuronal cells originate from the oligodendrocyte lineage (42.1%, Fig. 1a) and are segregated into 15 clusters (Fig. 2a). A recent study from our group showed that oligodendrocyte differentiation in the median eminence is nutritionally regulated and plays a role in controlling energy balance^44^. HypoMap captures different stages of oligodendrocyte differentiation, from progenitor cells marked with *Pdgfra* and *Ng2*, to differentiating cells with decreasing *Bmp4* and *Olig2* expression, and mature oligodendrocytes with increased expression of myelination genes, such as *Mbp* and *Mog* (Supplementary Table 5).

The second largest non-neuronal subtree consists of astrocytes and ependymal cells (39.1%). Astrocytes with high levels of *Slc1a2* and *Gjb6* were divided into nine clusters (Fig. 2a). These include a cluster of reactive astrocytes (C66-54: Lgals3.Astrocytes), marked with high expression of *Gfap* and *Lgals3*. Neighboring the astrocytes are *Vim*-expressing ependymal cells and tanycytes (four subclusters each), both of which form tight junctions around the third ventricle and regulate its permeability. Ependymal cells are marked with high expression of *Ccdc153*, and tanycytes have high expression of *Col23a1*, *Fgf10*, and *Crym*. The tanycyte subclusters are consistent with previous division into alpha and beta tanycytes by Campbell et al.^5^ (Fig. 1b) and the Tany-seq atlas^45^. We also identified a small cluster of hypendymal cells from the subcommissural organ, marked by expression of *Spp2*. HypoMap also captures a large cluster of *Ly86*-expressing microglia (Fig. 2a), which could be further divided into ten clusters (Fig. 2a). Nutritional challenges, such as a high-fat diet (HFD), are known to regulate the activity of non-neuronal cells^46–48^ in the hypothalamus; it would be of interest to investigate how such perturbations will affect these populations in future studies.

### Single-nucleus sequencing of the mouse hypothalamus

**Fig. 4:**
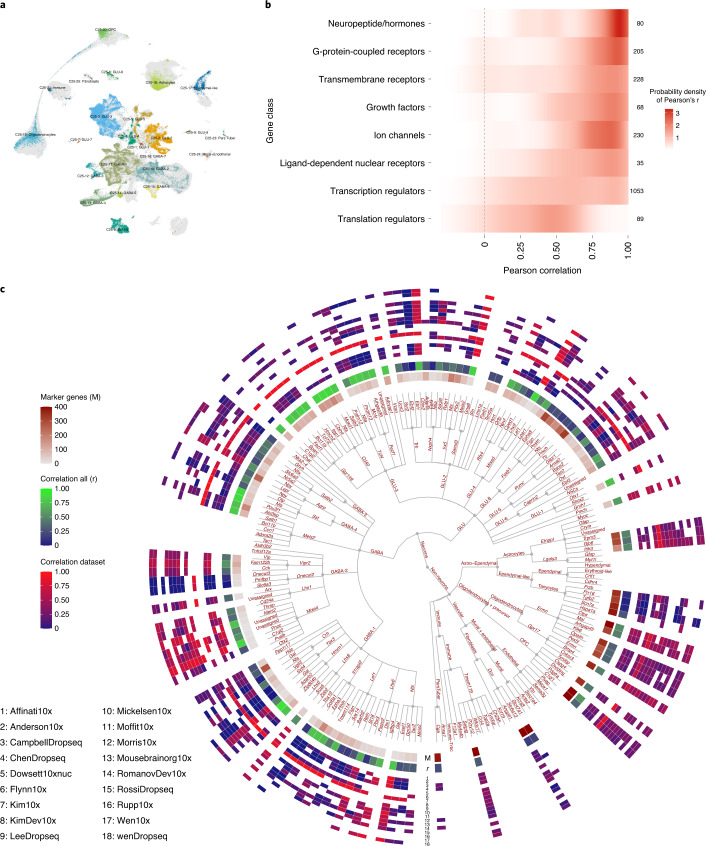
Comparison of nucSeq and single-cell data. **a**, UMAP visualization of the nucSeq data colored and annotated by cluster level 3 (C25) on all HypoMap cells (gray), demonstrating that the nucSeq data are evenly integrated in HypoMap. **b**, Heatmap of per-gene correlation (Pearson’s *r*) between sc-seq and nucSeq. Each row shows the density (color) of all genes in a specific gene class (number of genes shown on the right). Also see Supplementary Table 9. **c**, Heatmap of cluster-level correlation shown on the hierarchical tree of neuron clusters. For each cluster, the marker genes (M, number depicted in inner heatmap in red) were used to calculate Pearson’s *r* between all sc-seq and nucSeq data (middle heatmap in blue–green) or between individual HypoMap datasets and nucSeq (outer heatmap in blue–red). If there were fewer than ten cells per cluster and dataset, the comparison was omitted (white). Source data

We performed nucSeq from mice that either were subjected to an overnight fast or were ad libitum chow fed^49^. The sequencing yielded data for 36,626 nuclei, which were integrated into HypoMap. Despite the difference in techniques, the nuclei are distributed throughout HypoMap with little evidence of technical artifact (Fig. 4a). At C185, nucSeq covers 163 out of 185 clusters (Supplementary Table 7). There is an under-representation of some cell types, particularly those originating from the POA, SCN, and PVH.

We next examined the difference in gene expression between nucSeq and all sc-seq datasets included in HypoMap. Consistent with recent findings^50^, most genes showed a positive correlation (Fig. 4b). Notably, we found that neuropeptides/hormones, G-protein-coupled receptors (for example, *Glp1r*), ion channels (for example, *Kcnq3*), and nuclear receptors (for example, *Nr5a1*) all have high average Pearson coefficients (*r*) of 0.789, 0.743, 0.736, and 0.689, respectively. Growth factors (for example, *Vgf*) also showed a high correlation of *r* = 0.737. Other protein classes, such as transcriptional and translation regulators, have lower *r* values of 0.544 and 0.358, respectively.

When we examined the gene expression correlation on a per-cluster basis (see Methods), we found that the expression profiles in nucSeq showed a high correlation with the Kim et al.^32^ dataset, whereas the correlation with earlier Drop-seq datasets, such as Campbell et al.^5^, was poorer, despite an emphasis on the same hypothalamic regions (Fig. 4c and Supplementary Table 9). The overall correlation of sc-seq and nucSeq per cluster, unsurprisingly, varies between clusters (middle ring in the heatmap in Fig. 4c). Nevertheless, these experiments highlight the overall concordance between the sc-seq and nucSeq results, and thus the feasibility to use them for unified data integration.

### Inferring state-dependent neuron activation from nucSeq data

**Fig. 5:**
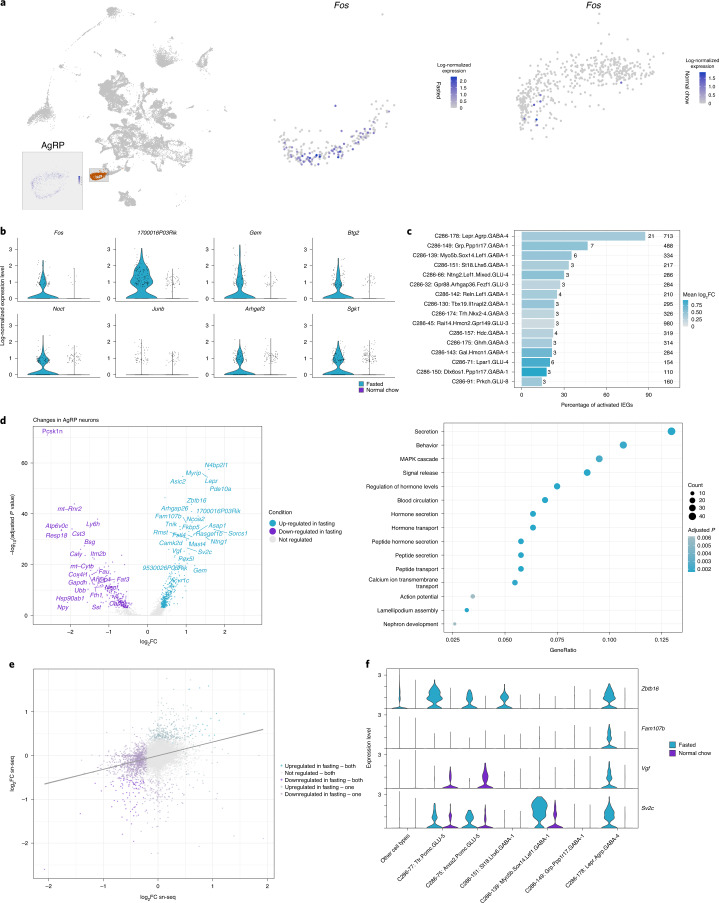
Transcriptional changes induced by fasting. **a**, *Fos* is increased in nucSeq AgRP neurons after fasting. Left, UMAP plot depicting C66-46: Agrp.GABA-4 in HypoMap. The inset shows *Agrp* expression in nucSeq cells from C66-46: Agrp.GABA-4. Right, UMAP plots of the same cells showing *Fos* expression in fasted and ad-libitum-fed conditions. The changes in nucSeq AgRP neurons after fasting were strong enough to cause a shift in the cluster. **b**, IEGs with high log_2_(fold change) (log_2_FC) in AgRP neurons. The violin plots show the per-cell expression between conditions. **c**, Neuron clusters activated by fasting. The bar plot depicts the percentage of significantly up-regulated IEGs in the fasted state over the total number of expressed IEGs (left number, based on presence in at least 10% cells of clusters in either condition). AgRP neurons are strongly activated, as indicated by the high number of changing IEGs. The bars are colored by mean log_2_FC, and the number of cells in each cluster is shown on the right. **d**, Transcriptional changes in AgRP neurons induced by fasting. In the volcano plot (log_2_FC versus adjusted *P* values from a (two-sided) Wald test), differentially expressed genes (DEGs) are highlighted. The dot plot shows Gene Ontology (GO) terms enriched in up-regulated genes. The *P* values are based on a hypergeometric test from an over-representation analysis and were corrected using false discovery rate (FDR). **e**, Comparison of transcriptional changes in AgRP neurons between nucSeq and Campbell et al.^5^ data. The scatter plot of log_2_FCs is colored by DEGs in either dataset. **f**, Per-cell expression levels of selected DEGs between conditions. For each gene, the expression is shown across multiple activated cell types as well as POMC neurons and a reference containing all remaining cells. *P* values of DEGs were obtained by Wilcoxon rank-sum tests and were adjusted for multiple comparisons using Bonferroni correction. See also Supplementary Tables 10 and 11. Source data

**Extended Data Fig. 7:**
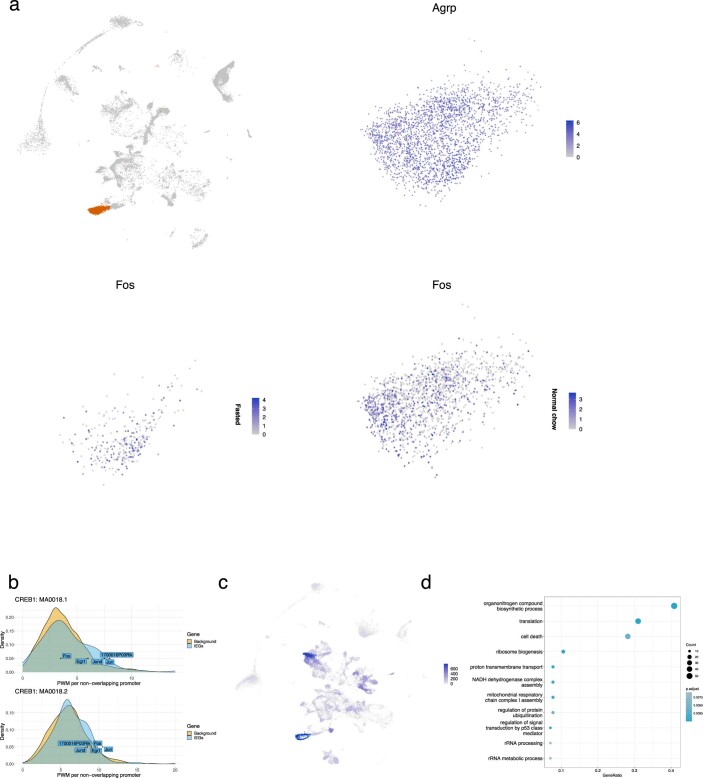
Neuronal changes after fasting. **a**, there is no change in *Fos* expression in Campbell *et al*. AgRP neurons after fasting. Left: UMAP plot depicting C66-46: Agrp.GABA-4 in HypoMap. The inset shows *Agrp* expression in Campbell *et al*. cells from C66-46: Agrp.GABA-4*C66-46: Agrp.GABA-4*. Right: UMAP plots of the same cells showing *Fos* expression in fasted and *ad libitum* fed states. **b**, CREB1 transcription factor binding site (TFBS) detection in IEG promoters compared to 1000 randomly sampled background gene promoters and IEG promoters. *1700016P03Rik* contains multiple putative CREB1 binding sites in its promoter sequence, indicating it could play a role as an immediate early gene. **c**, Number of differentially expressed genes (DEG) after fasting in each cluster shown on the UMAP of nucSeq data. **d**, Enrichment of DEGs that were found in at least 20% of all clusters (155 genes) in ‘biological process’ gene ontology (GO) terms. The pvalues are based on a hypergeometric test from an over representation analysis and corrected using false discovery rate (fdr). Globally regulated genes are enriched in translation related terms and ‘cell death’. Source data

In sc-seq, the *Fos* signal is often unreliable owing to the short half-life of mRNA, as well as artifacts originating from the enzymatic cell-dissociation procedure^17^, which was reflected in the absence of difference in *Fos* between fasted and ad-libitum-fed states in Campbell et al.^5^ (Extended Data Fig. 7a). In the nucSeq dataset, we detected an upregulation of *Fos* in AgRP neurons (C66-46: Agrp.GABA-4) in the fasted state (Fig. 5a), indicative of increased neuronal activity^17,32^. In addition, we also found that the effect of fasting is strong enough to influence the clustering of AgRP neurons (Fig. 5a). Thus, in the following analysis, we used the higher-level classification C66-46: Agrp.GABA-4 for AgRP neurons and C286 for all other clusters. We further examined other immediate early genes (IEGs) in AgRP neurons and found that they also exhibited comparable fasting-induced expression (Fig. 5b). We therefore aggregated the response of all IEGs (see Methods) and identified 15 additional neuronal clusters that showed increased neuronal activity in the fasting state (Fig. 5c and Supplementary Table 10). The effect was strongest in C66: Agrp.GABA-4, with an upregulation of ~90% of expressed IEGs. This is followed by two other ARC clusters: C286-149: Grp.Ppp1r17.GABA-1 and C286-139: Myo5b.Sox14.Lef1.GABA-1 (Fig. 5c). Here, we conducted the differential analysis on C286 with high granularity, because we observed that changes were restricted to specific subclusters, while other daughter nodes under C185-88: Sox14.Lef1.GABA-1 showed no difference (Fig. 5c). *Lef1* has previously been shown to be crucial for energy homeostasis^51,52^, and the identification of *Lef1*-expressing subclusters pinpoints the specific cellular subtypes and their molecular characteristics for future studies.

Next, we examined DEGs in the fasted state (Extended Data Fig. 7c and Supplementary Table 11). The top cluster was C66: Agrp.GABA-4 neurons, with 797 DEGs. This was followed by C286-45: Rai14.Hmcn2.Gpr149.GLU-3, with 757 DEGs. There were 172 DEGs detected in C286-149: Grp.Ppp1r17.GABA-1 and 62 DEGs in C286-139: Myo5b.Sox14.Lef1.GABA-1 neurons. Gene Ontology enrichment analysis revealed that the majority of the DEGs in AgRP neurons were involved in hormone secretion and release, and responses to neuronal activity changes (Fig. 5d). We also compared DEGs after fasting between the nucSeq and Campbell et al.^5^ sc-seq datasets, and found that DEGs from both datasets were positively correlated, with *r* = 0.3618 (*P* < 2.2× 10^–16^) (Fig. 5e). Among the top DEGs in AgRP neurons and other activated cell types were *Zbtb16*, *Fam107b*, *Vgf*, and *Sv2c* (Fig. 5f), which exhibited varying patterns of expression changes. For example, *Zbtb16 w*as up-regulated in many other cell types, aside from AgRP neurons, suggesting that it has a more global role. We also examined all DEGs across all clusters: 1,738 genes were significantly regulated in at least one cluster, and 33 and 155 of the DEGs were found to be significantly regulated in at least 50% or 20% of all clusters, respectively. The Gene Ontology enrichment analysis of overlapping DEGs between at least 20% of all clusters revealed pathways including protein translation and cell death (Extended Data Fig. 7d).

### Evaluation with Smart-Seq+Fluidigm C1 and bacTRAP datasets

**Fig. 6:**
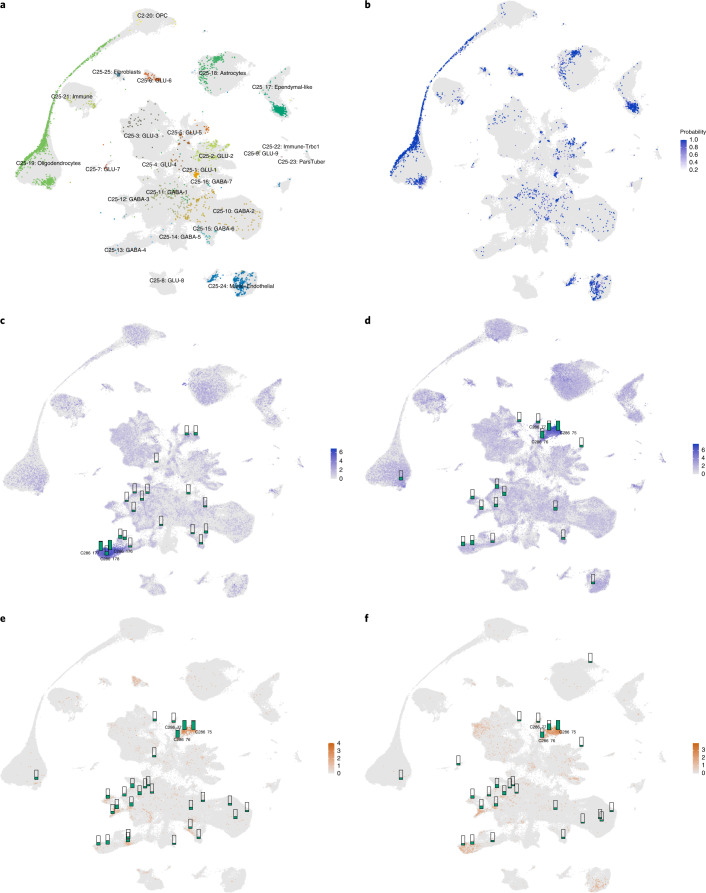
Projection of new data. **a**, HypoMap UMAP colored by cluster level 3 (C25) and overlaid with the projected ‘locations’ of cells from Romanov et al.^20^. Even clusters represented by only few cells in the query dataset can be accurately embedded into the reference. **b**, Probability scores (see Methods) of projection accuracy of Romanov et al.^20^ cells from **a**. High scores indicate high confidence in the projection, which is the case for most cells. **c**–**f**, Enrichment of bacTRAP signatures of specific neuronal population on HypoMap clusters using rank-biased overlap (RBO). RBO scores per cluster (C286) are shown as small bars relative to the highest score of each signature enrichment. The UMAP shows the expression level of the marker gene used in the bacTRAP experiment in HypoMap. In **e** and **f**, it shows the cells that express the combination of marker genes in orange (square root of product of expression levels). The corresponding cluster names for each unique ID in the figure can be found in Supplementary Table 3. **c**, AgRP^Cre^ neurons are enriched in C66-46: Agrp.GABA-4 subclusters. **d**, Pomc^Cre^ neurons are highly enriched in the C66-19: Pomc.GLU-5 subclusters, with medium scores in C66-46: Agrp.GABA-4. **e**, Pomc^Dre^Lepr^Cre^ neurons are most enriched in C286-75: Anxa2.Pomc.GLU-5, which expresses *Lepr*. **f**, Pomc^Dre^Glp1r^Cre^ neurons are most enriched in C286-77: Ttr.Pomc.GLU-5, which expresses *Glp1r*. Also see Supplementary Table 19. Source data

**Fig. 7:**
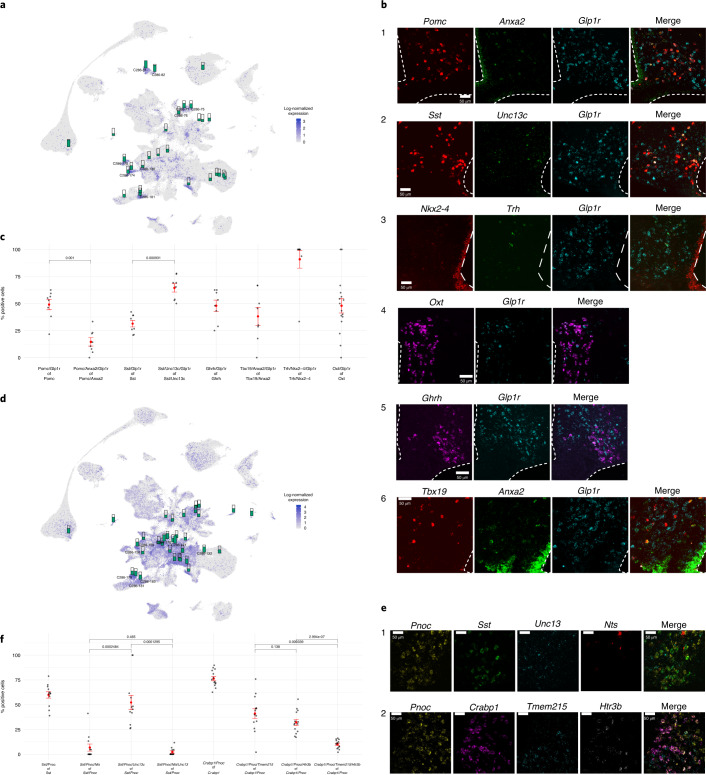
Validation of heterogeneous neuronal populations. **a**–**c***, Glp1r*-expressing cell types identified the hypothalamus. **a**, The Glp1r^Cre^ bacTRAP signature is enriched in multiple hypothalamic cell types, mostly corresponding to the *Glp1r* expression in HypoMap. **b**,**c**, RNAscope of *Glp1r* together with specific markers of neuron clusters identified using *Glp1r*-bacTRAP in (**a**). Representative images (**b**) and quantification shown as the percentage *Glp1r*-positive cells identified by marker gene expression (**c**). Points refer to individual sections, in total 4 rostral and 4 caudal ARC sections from 4 mice were included for each experiment (0 rostral and 8 caudal for *Tbx19*- plus *Anxa2* and 16 PVH sections for *Oxt*). Mean ± s.e.m,: Pomc: 49.03 ± 4.77; *Pomc*/*Anxa2:* 14.56 ± 3.89; *Sst*: 31.39 ± 3.06; *Sst*/*Unc13c*: 64.46 ± 3.88; *Ghrh*: 47.93 ± 5.19; *Tbx19*/*Anxa2*: 38.13 ± 8.3; *Trh*/*Nkx2-4*: 90.89 ± 8.26; *Oxt*: 47.94 ± 6.73. **d**–**f**, *Pnoc*-expressing cell types identified in the hypothalamus. **d**, The Pnoc^Cre^ bacTRAP signature is enriched in multiple hypothalamic cell types, and covers only a subset of *Pnoc*-expressing cell types in HypoMap. **e**,**f**, RNAscope of *Pnoc* and marker genes of selected ARC neuronal cell types based on *Pnoc*-bacTRAP and gene expression in (**d**). **e**,**f**, Representative images (**e**) and quantification (**f**) of *Pnoc* and *Sst* or *Crabp1* co-expressing subclusters. Points refer to individual sections, in total 14 ARC sections along the rostral-caudal axis from 4 mice were included for each experiment. Mean ± s.e.m: *Sst*/*Pnoc*: 59.9 ± 3.24; *Sst*/*Pnoc*/*Nts*: 6.78 ± 3.56; *Sst*/*Pnoc*/*Unc13c*: 52.28 ± 7.17; *Sst*/*Pnoc*/*Nts*/*Unc13*: 2.29 ± 1.1; *Crabp1*/*Pnoc*: 76.35 ± 2.2; *Crabp1*/*Pnoc*/*Tmem215*: 41.02 ± 4.81; *Crabp1*/*Pnoc*/*Htr3b*: 32.18 ± 3.04; *Crabp1*/*Pnoc*/*Tmem215*/*Htr3b*: 10.02 ± 1.17. In all dot plots, the red point depicts the mean and red error bars the s.e.m. of all sections. We used a two-sided Wilcoxon rank-sum test (multiple testing correction with Benjamini–Hochberg) to test for differences between the means of relevant groups and added the resulting *P* values to the quantification in (**c**) and (**f**). Source data

To further evaluate the utility of HypoMap, we projected external datasets onto the reference map from (1) a hypothalamic sc-seq dataset by Romanov et al.^20^ and (2) bulk bacTRAP RNA-seq of specific neuronal populations across different levels of cellular heterogeneity (Figs. 6 and 7 and Supplementary Tables 13–18).

Romanov et al.^20^ reported one of the earliest sc-seq datasets generated for the hypothalamus using traditional Smart-Seq+Fluidigm C1 (ref. ^20^). We successfully projected cells from this dataset onto HypoMap (Fig. 6a and Supplementary Table 12). At C185, cells were assigned to 125 of 185 clusters, although only 41 clusters contained 10 or more mapped cells. Most cell type labels were projected with high confidence (Fig. 6b). Consistent with the original study, the majority of cells were oligodendrocytes (41% of all cells). Among neuronal clusters, *Shox2*-expressing neurons (C185-1: Shox2.GLU-1) (5.6%) were the most common assignment, containing 163 cells, which were mostly left unclassified before (Fig. 6a).

Further inspection of the original annotations demonstrated that the inferred cluster labels are an accurate reflection of the true identity of these cells. For example, 12 of 23 cells annotated as ‘GABA 13 (Galanin)’ were projected onto *Gal*- and *Th*-expressing C185-91: Gal.Hmcn1.GABA-1 cluster of HypoMap, and others onto closely related clusters and other *Gal*-expressing clusters. Furthermore, HypoMap enhanced the stratification of the original annotations: out of 27 cells annotated as ‘GABA 7 (Pomc +/–),’ only 7 are *Pomc*-positive and 5 of these cells were mapped to the Pomc.GLU-5 cluster, whereas all *Pomc*-negative cells were relocated to other HypoMap clusters.

bacTRAP RNA-seq is a useful tool to obtain marker-gene-specific molecular expression profiles with a great sequencing depth and the possibility to compare between different conditions^53^. We generated bacTRAP of specific hypothalamic cell types previously shown to be involved in energy homeostasis, that vary in their heterogeneity and anatomical distribution. To this end, we first crossed mice expressing Cre-recombinase in either AgRP, POMC, PNOC, or GLP1R neurons with mice allowing for Cre-dependent expression of ribosomal protein L10a fused with green fluorescent protein^6^. We then mapped these bacTRAP datasets onto HypoMap to examine the concordance of gene expression and determine whether HypoMap could be used to deconstruct the cellular composition in bacTRAP.

We first focused on bacTRAP obtained from AgRP and POMC neurons, which are relatively homogeneous cell types, and we further explored more heterogeneous data obtained from PNOC and GLP1-R neurons. We selected genes that were significantly enriched in the immunoprecipitation (IP) samples compared to the control samples of the bacTRAP datasets to define cell type signatures (see Supplementary Tables 13–18 for all DEGs). Subsequently, we compared HypoMap cluster markers and the signature fold changes using rank biased overlap (RBO), which allows the comparison of ranked sets with more weighting for the top part of the list that contains the most relevant markers^54^.

In Figure 6c, we highlight the AgRP bacTRAP signature enrichment as small bars on top of the UMAP plot, showing the expression of *Agrp* in the sc-seq of HypoMap. This demonstrates that the AgRP signature is mapped successfully onto the cluster containing AgRP neurons in HypoMap (C66-46: Agrp.GABA-4), and indicates that the sc-seq integration grouped all AgRP neurons into one branch of the tree, and therefore could be used for further interpretation (Fig. 6c and Supplementary Table 19). There were other enriched ARC clusters with lower enrichment scores, indicating that they share some of the marker genes with AgRP neurons.

Next, we analyzed the POMC bacTRAP data (Fig. 6d): the C286-75: Anxa2.Pomc.GLU-5 cluster at level C286 (score = 0.256) was more enriched than the other *Pomc*-expressing clusters, C286-77: Ttr.Pomc.GLU-5 (score = 0.197) and C286-76: Glipr1.Pomc.GLU-5 (score = 0.185), an effect that is at least partly driven by the higher abundance of canonical marker genes for POMC neurons, such as *Cartpt* (Supplementary Table 5). In addition, we observed low-grade mapping of the POMC bacTRAP data onto *Agrp*-expressing clusters (Fig. 6d), and the enrichment score for all other clusters was lower.

We recently found that leptin receptor (*Lepr*)- and *Glp1r*-expressing POMC neurons showed little overlap and have distinct molecular signatures and functions^6^. In this study, we profiled these two POMC subpopulations using intersectional Cre/Dre-dependent targeting. This approach builds on the use of POMC^Dre^ as well as Lepr^Cre^ and Glp1r^Cre^ transgenic mice. In contrast to the POMC^Cre^ transgenic model, POMC^Dre^ expression exhibits a progressive increase in recombination later in adulthood, thereby circumventing the developmental marking of AgRP neurons^6^. Analysis of the intersectional POMC bacTRAP gene signatures revealed that the *Pomc*-*Lepr* signature overlaps mostly with C286-75: Anxa2.Pomc.GLU-5 (score = 0.415), but scores lower in other *Pomc*-expressing clusters (Fig. 6e), as previously validated functionally^6^. This is consistent with the expression of *Pomc* and *Lepr* depicted in the UMAP of Figure 6e (only cells expressing both genes are highlighted). The enrichment of the *Pomc*-*Glp1r* signature was highest in C286-77: Ttr.Pomc.GLU-5 (score = 0.325), which is concordant with the expression of *Glp1r* and *Pomc* in HypoMap (Fig. 6f). The high enrichment score in C286-75: Anxa2.Pomc.GLU-5 (score = 0.299) was likely driven by the high expression of marker genes, such as *Pomc* and *Cartpt*, similar to POMC-only bacTRAP.

### Validation of predicted *Glp1r*-expressing neurons

Next, we turned to the more heterogeneous *Glp1r-* and *Pnoc-*expressing neurons. Again, we utilized the bacTRAP signature for *Glp1r*-expressing hypothalamic cells to investigate their heterogeneity in HypoMap. At C286, the highest enrichment of *Glp1r* bacTRAP was found in the *Avp*- and *Oxt*-expressing neurons C286-81: Ebf3.Caprin2.GLU-6 (score = 0.19) and C286-82: Oxt.Caprin2.GLU-6 (score = 0.166), both from the PVH, consistent with previous findings^55^ (Fig. 7a). Interestingly, *Glp1r*-expressing POMC neurons (C286-77: Ttr.Pomc.GLU-5)^6,24^ were enriched, with a lower score of 0.119. We identified four additional populations on the basis of high *Glp1r* expression and enrichment score: C286-175: Ghrh.GABA-3, C286-174: Trh.Nkx2-4.GABA-3, C286-181: Il1rapl2.Otp.Sst.GABA-4, and C286-130: Tbx19.Il1rapl2.GABA-1 (Fig. 7a and Supplementary Table 19).

We performed multiplexed single-molecule in situ hybridization to identify the spatial distribution of *Glp1r*-expressing populations (Fig. 7). Using probes specific to *Glp1r, Pomc*, and *Anxa2* for the Ttr.Pomc and Anxa2.Pomc subclusters, we found that although 49% of all POMC^ARC^ neurons expressed *Glp1r*, only 14.6% co-expressed *Pomc* and *Anxa2* (Fig. 7b,c). For the SST^ARC^ neurons, we used probes against *Sst* and *Unc13c* to target the C286-181: Il1rapl2.Otp.Sst.GABA-4 cluster and found that 64.5% of *Sst*/*Unc13c*-positive cells expressed *Glp1r* (Fig. 7b,c). When we used *Sst* as the only marker gene, the percentage of *Glp1r*-positive cells decreased to 31.4%, indicating that C286-181: Il1rapl2.Otp.Sst.GABA-4 is indeed the most relevant SST^ARC^ subtype (Fig. 7b,c).

*Ghrh* is a distinct marker of C286-175: Ghrh.GABA-3 neurons, which control growth via the growth hormone (GH)–insulin-like growth factor 1 (IGF1) axis^56^. We found that 47.9% of *Ghrh*-expressing cells expressed *Glp1r* (Fig. 7b,c). However, GLP-1 action has not been investigated in these neurons, and these findings offer the possibility that they may serve as an integrator in the adaptation of metabolism and growth.

**Extended Data Fig. 8:**
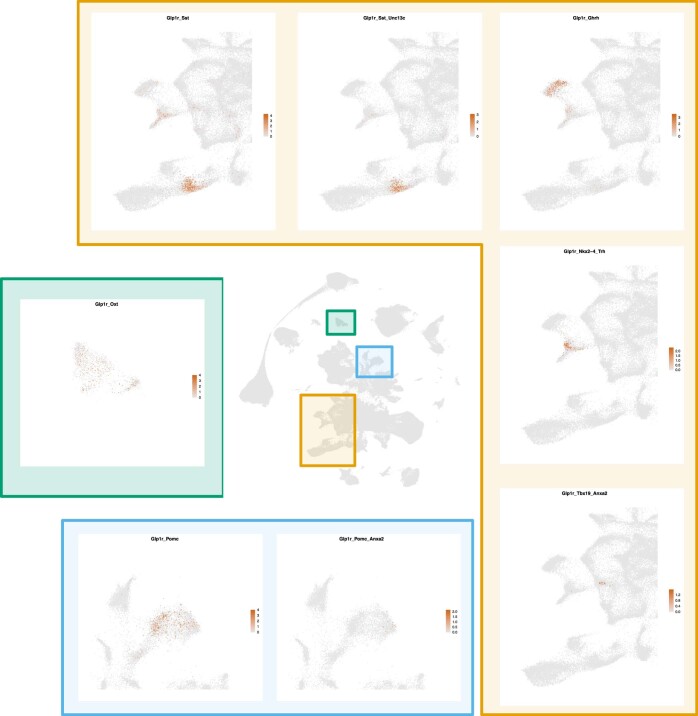
Gene expression of marker genes used for Glp1r RNAscope validation. For each marker combination that was quantified together with *Glp1r* (see also Fig. 7a-c), double or triple positive cells are shown on UMAP subsets around the relevant cell types. The color scale is based on the square root of the product of expression levels. The colored rectangles in the grey reference in the central UMAP show which parts of the complete HypoMap are shown in the subsets. For most cell types the gene combinations are highly specific, especially compared to their neighboring cells. However, for *Tbx19*/*Anxa2* only very few *Glp1r* cells exist in the single cell data. Source data

*Anxa2* is expressed in C286-130: Tbx19.Il1rapl2.GABA-1. Using probes specific for *Anxa2* and *Tbx19*, we found that 38.1% of *Anxa2*- and *Tbx19*-positive cells expressed *Glp1r* (Fig. 7b,c), which was markedly higher than the number of triple-positive cells identified in sc-seq (Extended Data Fig. 8). *Trh* and *Nkx2-4* were used to distinguish C286-174: Trh.Nkx2-4.GABA-3 from other *Trh*-expressing cells. Although, we detected only a few *Trh*-positive cells in ARC, and not all of them were *Nkx2-4*-positive, we found a striking overlap with *Glp1r* expression: 90.9% of *Trh*- and *Nkx2-4*-expressing cells were *Glp1r*-positive (Fig. 7c). Last, we examined the expression of *Glp1r* in *Oxt*-expressing cells in the PVH, which had the strongest bacTRAP enrichment; 47.9% of the *Oxt*-expressing cells were *Glp1r*-positive (Fig. 7C), consistent with the previous findings^38^.

### Validation of predicted *Pnoc*-expressing neurons

*Pnoc* is widely expressed in the hypothalamus, thus the molecular signature obtained through bacTRAP reflects the heterogeneity of this diverse population (Fig. 7d). Projecting *Pnoc* bacTrap onto HypoMap resulted in lower enrichment score overall than that of the AgRP and POMC bacTRAP, but resulted in more enriched clusters (Fig. 7d). Many of these clusters express *Pnoc* at high levels in HypoMap, indicating that *Pnoc* bacTRAP is identifying these cell types correctly. At tree level C286, the highest enrichment was found in C286-171: Tac2.Nts.GABA-1 (score = 0.13). Interestingly, some clusters, such as C286-85: Nts.Foxb1.GLU-8 from the mammillary region, also express *Pnoc* (55.4% of cells), but were not enriched in bacTRAP (score = 0.005), suggesting that the IP might not have captured some of these *Pnoc*-expressing neurons. Conversely, C286-84: Pmch.GLU-7 (score = 0.088) and histamine-producing C286-157: Hdc.GABA-1 (score = 0.07) from the tuberomammillary nucleus were enriched in *Pnoc*-specific bacTRAP, but did not show high *Pnoc* expression in HypoMap (17.5% and 16.2% of cells, respectively).

**Extended Data Fig. 9:**
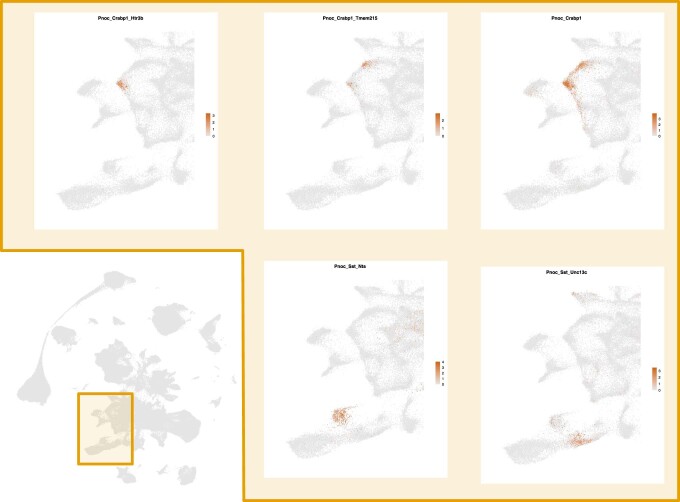
Gene expression of marker genes used for *Pnoc* RNAscope validation. For each marker combination that was quantified together with *Pnoc* (see also Fig. 7e-f), double or triple positive cells are shown on UMAP subsets around the relevant cell types (See Extended Data Fig. 8 for details). Source data

Owing to the heterogeneity of *Pnoc*-expressing cell types, we focused on clusters from ARC. Again, some cell types such as C286-175: Ghrh.GABA-3 expressed *Pnoc* at moderate levels (23.1%), but were not enriched in bacTRAP (score = 0.018). Among cell types that were concordant between bacTRAP and sc-seq were multiple *Sst*-expressing clusters and two *Crabp1-*expressing clusters (Fig. 7d and Supplementary Table 19). We validated *Sst*- and *Crabp1*-positive populations, including multiple subclusters of *Unc13c*-expressing C185-118: Otp.Sst.GABA-4 (28.3% *Pnoc*-positive) and *Nts*-expressing C185-117: Npy.Sst.GABA-4 (65.9% *Pnoc*-positive) populations, which were also enriched in bacTRAP. Using RNAScope, we further validated that a large proportion of *Sst*^ARC^ cells co-expressed *Pnoc* (59.9%) (Fig. 7e,f). Similarly, 52.2% of the *Unc13c*-and *Sst*-positive cells also expressed *Pnoc*. We found a very small overlap between *Nts* and *Unc13c* expression in *Pnoc*- and *Sst*-positive cells in the ARC (2.3%), thus confirming the segregation suggested by HypoMap (Extended Data Fig. 9). However, very few cells expressed *Nts* in the *Pnoc*-and *Sst*-positive cells (6.7%) (Fig. 7e,f).

The *Htr3b*-expressing C286-158: Vgll3.Tbx3.GABA-1 (39.0% *Pnoc*-positive in HypoMap) and the closely related *Tmem215*-expressing C286-159: Crabp1.Sytl4.Tbx3.GABA-1 (26.3% *Pnoc*-positive in HypoMap) were enriched in bacTRAP, with scores of 0.078 and 0.047, respectively (Fig. 7e,f and Extended Data Fig. 9). Using RNAScope, we validated the expression of *Pnoc* in *Crabp1*-expressing cells (76.35%), as well as the subcluster of *Tmem215*-expressing cells (41% of *Pnoc*- and *Crabp1*-positive were *Tmem215*-positive) and *Htr3b*-expressing cells (32.2% of *Pnoc*- and *Crabp1*-positive were *Htr3* -positive).

## Discussion

HypoMap, a harmonized transcriptomic reference map of the murine hypothalamus, faithfully integrates 18 single-cell sequencing experiments that cover almost all hypothalamic regions. It allows efficient interpretation of new datasets by harmonizing cell type labels, identifying previously unannotated populations, and inferring anatomical localization. We demonstrated that data integration is an essential step towards comparability of sc-seq/nucSeq studies and that choosing a reliable data integration algorithm can be challenging^10^. We used well-defined neuronal populations to ensure that the algorithm did not over-correct, by removing the boundaries between truly distinct cell types while inter-mixing datasets sufficiently.

We chose scVI^15^ as the best purity-preserving integration method, which also offers a ‘future proof’ ability to map new datasets to the reference. Harmony, Scanorama, and Combat also performed well, when tuned properly, especially when using a large number of latent variables^12,14,16^. Overall, while computational metrics can assist in determination of an optimal integration method, these should be combined with careful evaluation and experimental validation.

We showed that nucSeq covers many of the clusters in HypoMap (163 of 185). Comparing the transcriptomic profiles of sc-seq and nucSeq highlighted a dataset-related bias: it correlates better with some datasets (for example, Kim et al.^32^), which could be related to the single-cell technology used (that is, Drop-seq versus 10x). However, because some 10x-based datasets (for example, Moffit et al.^30^ and Mickelsen et al. (Flynn10x)^27^) show a lower correlation, this may not be the only factor. Differences also exist between cell types; for example, the *Trh*-expressing cluster C185-12: Ebf1.Trh.GLU-2 showed a markedly lower *r* value of 0.017 (across all datasets) than that of its sister-cluster C185-11: Cbln2.Trh.GLU-2 (*r* = 0.789) (Fig. 4c). This divergence could be related to differences in RNA species measured between sc-seq and nucSeq.

Changes in IEGs can be used to infer neuronal activity^17,32^. Here, AgRP neurons are known to be activated during fasting^57^ and are used as positive controls. No other cell type showed an effect similar in strength compared to AgRP neurons, but some, such as C286-149: Grp.Ppp1r17.GABA-1 and C286-139: Myo5b.Sox14.Lef1.GABA-1, could be interesting targets for future studies. When comparing cells from fasted and ad-libitum-fed mice, we detected DEGs in many cell types. Interestingly, many of the DEGs are common across multiple clusters. This indicates that these changes may not be cluster specific, but might follow a common program (for example, protein translation) in response to a change of the metabolic state.

To demonstrate the utility of HypoMap, we showed that the underlying scVI model is able to project new hypothalamic sc-seq data, such as the Smart-Seq+Fluidigm C1 data by Romanov et al.^20^ onto the existing reference, thereby allowing quick and reliable annotation of cells in new datasets.

We also attempted to project bacTRAP bulk RNA-seq data of known neuronal populations onto HypoMap, to demonstrate that HypoMap was able to segregate these neurons over a wide range of cellular complexities and heterogeneity. Importantly, the POMC^Cre^ transgenic-based translational profiling not only faithfully captured bona fide *Pomc*-expressing cells, but also detected the AgRP neuron cluster, which expressed the POMC^Cre^ transgene developmentally and had thus been lineage-traced in this approach, as previously described^58^. However, HypoMap was also able to differentiate distinct POMC neuron subpopulations, as revealed by selective ribosomal profiling of *Lepr*- and *Glp1r*-expressing POMC neurons via intersectional recombinase based targeting^6^. Our recent study using this system allowed us to unravel the microcircuit architecture and functional consequences of these heterogeneous cell groups^6^. Mapping of the more complex *Pnoc* and *Glp1r* bacTRAP datasets further illustrates how HypoMap can be successfully used to disentangle complex cell type mixtures. Compared with AgRP and POMC neurons, *Pnoc* and *Glp1r* neurons are less well characterized populations, and HypoMap offers an opportunity to better define these diverse, physiologically relevant neuron subtypes.

Here, we validated six *Glp1r*-expressing cell populations in the ARC and other hypothalamic regions. GLP-1RA can activate POMC neurons and contributes to an acute anorexigenic effect^24^. Consistent with this, acute chemogenetic activation of *Glp1r-*expressing POMC neurons rapidly and potently suppressed food intake, but less so in *Lepr*-expressing POMC neurons^6^. Activation of *Glp1r*-expressing cells in the PVH has also been described to mediate an acute anorexigenic effect^55^, and HypoMap can identify these clusters: C286-82: Oxt.Caprin2.GLU-6 and C286-81: Ebf3.Caprin2.GLU-6, which were both validated by in situ hybridization. Integrating this information with the identification of additional *Glp1r*-expressing clusters in HypoMap may guide functional studies to further delineate the cells responsible for mediating the weight-reducing actions of GLP-1RAs. Candidate *Glp1r*-expressing clusters include *Trh* and *Nkx2-4* co-expressing populations and *Ghrh*-positive and *Tbx19*-positive populations. The enrichment observed in C286-75: Anxa2.Pomc.GLU-5 and C286-76: Glipr1.Pomc.GLU-5 was likely an artifact due to the high expression of POMC marker genes, as our previous mapping of *Pomc*-*Glp1r* bacTRAP data demonstrated.

We also validated multiple *Pnoc*-expressing populations in the ARC, such as the *Crabp1*-expressing clusters C286-158: Vgll3.Tbx3.GABA-1 and C286-159: Crabp1.Sytl4.Tbx3.GABA-1, as well as SST neurons (for example, Otp.Sst.GABA-4), which could mediate different effects than could other *Crabp1*-expressing cell types. Combining *Pnoc* bacTRAP with HypoMap allows the molecular characterization of the PNOC^ARC^ neurons at a high magnification and will enable further functional studies on their role in energy homeostasis. Our previous studies revealed that PNOC^ARC^ neurons are implicated in feeding behavior^26^. Thus, this cell population represents a promising new target for therapeutic interventions in obesity and warrants further studies on the role of defined subclusters via intersectional targeting.

We envision that HypoMap will be used to align newly generated datasets to annotate cell types. When complemented with studies of different hypothalamic regions, the full range of hypothalamic cell expression can be revealed. It has been shown that the brain, including the hypothalamus, is enriched for genes involved in heritability of body-mass index^59^. The unified cell-type annotation provided by HypoMap could further refine the enrichment analysis of genome-wide association studies of traits implicated in energy homeostasis. We expect that future versions of HypoMap will be extended by adding data from human and non-human primates, which will further increase the power of translational analyses.

HypoMap is limited only by the data we used to build it. Notably, some regions, such as the zona incerta, are underrepresented. We will continue to update HypoMap on a regular basis in order to incorporate new datasets and make it available to the scientific community.

It should be noted that we chose to not correct the count-level data, as we found that the count correction was often ‘smoothing’ differences between clusters too strongly, thereby limiting downstream biological interpretation. However, this also means that gene expression queries to HypoMap still represent a mixture of different datasets and technical modalities.

In summary, we systematically integrated sc-seq/nucSeq datasets into HypoMap, a unified single-cell reference of the murine hypothalamus. HypoMap can serve as a basis for functional studies that further define energy-sensing neurons, such as GLP1-R and PNOC neurons. We found that nucSeq is a reliable alternative to sc-seq, and is able to capture similar cell types with a good comparability. nucSeq from snap-frozen cells is able to preserve neuronal activation markers, which makes it an attractive modality for functional studies.

## Methods

### Dataset download

An overview of all datasets included in the reference map is available in Supplementary Table 1. Sequence reads from 17 publicly available datasets were downloaded using ‘fastq-dump’ from the SRA toolkit (version 3.0.0), with the exception of (1) GSE132355, GSE113576, GSE167927, and SRP135960, for which the original 10X BAM files were downloaded and used to regenerate fastq files via the ‘bamtofastq’ command from the Cellranger software (version 6.0.1, 10X Genomics); and (2) Kim et al.^32^, for which the original reads could not be found and the count table was used instead.

### Sequence alignment, UMI and gene count

For 10X datasets (including nucSeq), Cellranger Version 6.0.1 (5.0.1 for nucSeq) was used to (1) map sequence reads to the mouse genome GRCm38 (mm10); and (2) perform the UMI and gene-level counts against Ensembl gene model V100, with *Gm28040* removed to recover *Kiss1* expression. The per-cell gene count tables (in HDF5) generated by the software were then used for downstream analyses.

For each Drop-seq library, the sequence alignment of the biological reads was performed using STAR 2.7.5 to the mouse genome GRCm38 to generate a BAM file containing mapped reads. Read 1, which contains the cell barcode (CBC)/UMI read was first split into two separate fastq files, one containing just the CBC and the other containing the UMI, using ‘fastx_trimmer’ from the FASTX Toolkit (version 0.013), and were then tagged (XC and XM, respectively) onto the mapped biological reads using Fgbio’s ‘AnnotateBamWithUmis’ command (Fulcrum Genomics, version 1.5.1). All the untagged reads were removed from annotated BAM file using Samtools (version 1.14), and PCR duplicates based on UMI (XM tag) were removed using Picard’s ‘MarkDuplicates’ command (version 2.22.2).

The deduplicated BAM file was then further annotated with genes using the gene model above (GRCm38, Ensembl V100 minus Gm28040) using ‘TagReadWithGeneFunction’ from Drop-seq tools (version 2.3), and digital gene expression was performed using ‘DigitalExpression’ command from Drop-seq tools, using a minimum number of transcript cut-off of 800 UMIs to generate a gene-level expression matrix for all cells detected, in a tab-delimited text format.

### Dataset quality control

We used the expression matrices of each datasets and ran a basic preprocessing using Seurat (version 4.0.2)^9^. Each dataset was further filtered to contain cells with a minimum of 1,000 UMIs per cell and a maximum fraction of mitochondrial genes of 0.1. We curated a common set of metadata features, including unique cell, sample and dataset-IDs, sample description (sex, mouse strain, experimental condition), dataset descriptions (technology, regional identities), and cell details (author annotations, UMI counts, cellcycle scores), among others. We harmonized the annotations of major cell types (’classes’) to be comparable across datasets, including: neurons, tanycytes, astrocytes, oligodendrocytes, ependymocytes, immune cells, vascular/endothelial cells, and fibroblasts. For the data from Kim et al.^32^, no raw read files were available, hence we used the UMI count matrices provided by the authors and subset the cells to those annotated by the authors as hypothalamic cells (removing doublets, low-quality cells, and other unannotated cells). After identifying within-dataset batches, we merged all datasets to one combined dataset as input for our integration pipeline.

### Batch identification

We considered datasets as independent datasets if they were split into multiple datasets by the original authors (for example Wen et al.^31^). For all individual datasets, an automated detection of batches was used: starting with a default processing using Seurat, consisting of log-normalization, feature detection (2,000 features), and principal component analysis (PCA), a low-dimensional embedding with 50 PCs was obtained. Afterwards, randomForest R package (version 4.6) (https://cran.r-project.org/package=randomForest) was used to predict the sample ID using the PCs as features and cells as observations. Using hierarchical clustering with spearman correlations *ρ* of the pairwise out-of-bag (oob) probabilities as distances, a clustering of samples was obtained. We iteratively merged the closest samples in the associated dendrogram and trained another random forest only on the currently merged samples. The entropy of the oob class probabilities, normalized by the logarithm of the total number of samples and averaged over all cells, was used to estimate how well samples are separated. An entropy close to 1 means high similarity of samples; decreasing entropy indicates that the random forest can tell the merged samples apart and that there could be a batch effect. Merging of samples was stopped when the averaged entropy over all (merged) samples fell below 0.9. We calculated the entropy (*H*) as: $H\left( X \right) = - \mathop {\sum }\limits_{i = 1}^n p\left( {X_i} \right) \times log(p(X_i))$, where *p*(*X*_*i*_) is the vector of class probabilities.

### Doublet removal

We used DoubletFinder (https://github.com/chris-mcginnis-ucsf/DoubletFinder)^60^ to identify doublets within each batch (as defined above), independent of all other data sets. We used 70 PCs and a fixed value for the variable numbers of artificial doublets (pN) of 0.25. For the neighborhood size (pK), we iterated over multiple values to find an optimal one for each batch but limited it to a maximum of 0.1. For 10x sc-seq data, we set the expected rate of doublets to 0.05; for Drop-seq data and pre-filtered data (for example, Kim et al.^32^) we set the expected rate of doublets to 0.01. Using the DoubletFinder predictions and preliminary clusters from the per-batch preprocessing, we excluded all clusters with a percentage of doublets above 70%.

After obtaining the final harmonized version of the data (see the next sections), we ran another round of manual doublet curation to identify potential doublet clusters that formed only after combining all cells. For this, we took advantage of defined signatures for major cell types and an exploratory clustering on the harmonized data. If cells of a cluster had a high average score for more than one cell type, the cluster was marked and subsequently removed. To avoid removing intermediate cell types, all marked clusters were evaluated manually before removal.

### Data integration

Please see the Supplementary Information for information on data integration and evaluation. This includes a description of the pipeline, an overview of evaluated integration methods, the metrics used for evaluation, and the tuning of the final scVI model.

### Cluster detection

Clustering was conducted using the Leiden algorithm^42^ for single-cell data in scanpy on the shared nearest-neighbor graph, as implemented in Seurat^9^ (*k* = 25 neighbors). We iteratively increased the clustering resolution starting from 0.001 up to 50 to obtain a range of different cluster levels. The first clustering level was manually defined on the basis of the known annotation for neurons and non-neuronal cell types, and a Leiden clustering with seven groups was chosen as the second level (segregating into GABAergic and glutamatergic neurons and five major non-neuronal cell groups). We manually adjusted some small problematic clusters (for example, PMCH-neurons that were grouped with non-neuronal cell types). For the following five levels, we selected Leiden clustering results with increasing granularity, aiming to roughly triple the number of included clusters with each level. In total, 7 clustering levels yielding between 2 and 680 clusters were generated. These clusters were subsequently combined into a clustering tree using the Multiresolution Reconciled Tree (mrtree)^43^ algorithm. We used the mrtree function from the original package (https://github.com/pengminshi/MRtree) with standard parameters. We did not cut the resulting tree at an optimal level, but instead used it as a representation of the complex subtypes within neuronal cell types. After determining marker genes distinguishing each cluster node from its siblings in the tree (as described below), we merged sibling clusters with less than 10 relevant markers (specificity > 1, see ‘Cluster annotation’) into one cluster node to avoid over-clustering.

For visualization of the tree we used the ggtree R package (version 2.4.2)^61^ (https://github.com/YuLab-SMU/ggtree). VMH neuron cluster comparisons were visualized using the sankeyNetwork function from the networkD3 R package (version 0.4) (https://cran.r-project.org/package=networkD3).

### Marker gene detection

We used the van Elteren test implementation for Seurat objects (https://github.com/KChen-lab/stratified-tests-for-seurat) to detect marker genes for each node of the cluster tree, comparing the current cluster either against all other nodes on the same level or against the sibling nodes to obtain a set of markers per cluster. We subset the tree into its neurons and non-neurons and calculated the global marker genes only versus other cells in the respective subtrees to restrict the marker sets to more relevant genes.

### Cluster annotation

We calculated a specificity score, *S*, per gene and cluster, *c*, as $S_{c,ref} = Average\;log_{2}Foldchange_{c,ref} \times \frac{{Pct_c}}{{Pct_{ref}}}$ which includes the foldchange of the average expression of cluster *c* and a set of reference cells (for example, all others), and the percentage (Pct) of expressing cells in cluster *c* and the reference. We used the specificity *S* to rank genes, and additionally the adjusted *P* values from the van Elteren test to reduce this ranking to potential marker genes. To determine the most relevant marker genes and to select a cluster name, each node (cluster) of the tree was visited from top to bottom. We ranked the most descriptive markers by calculating a score (*T*) for each cluster *c* as $T_c = S_{c,all} \times S_{c,siblings}/\mathop {{\max }}\limits_i \left( {S_{i,siblings}} \right)$, which includes the specificity compared with all other clusters, as well as only the sibling clusters and the specificity for all children clusters *i*. Additionally, we excluded pre-specified genes such as ‘mt-’ or ‘Rp’ and any genes that were already used as a name in the ancestor nodes. To incorporate a node’s parent cluster, the final cluster annotation was constructed by concatenating the node’s best marker gene with its parent’s name. If no siblings existed, no additional gene name was added. For the first three levels of the tree, we manually set the names of clusters on the basis of the previously curated major cell types (‘class’) and, in some cases, specific neuronal markers.

### Region annotation

In order to predict potential spatial locations of clusters, we combined the likely regions of origin based on the original dataset (if specific to certain subregions), with a prediction based on ISH expression values from the Allen Brain Atlas^62^ (https://mouse.brain-map.org/). We used the cocoframer R package (version 0.1.1) (https://github.com/AllenInstitute/cocoframer) to query the API and obtain ISH expression values for each voxel from the coronal sections.

For each voxel we used the ‘energy’ value to rank all probes. Then, for each neuronal cluster from level 6 of the HypoMap tree (C286), we selected all probes with matching marker genes (specificity > 4) and calculated the median rank in that voxel as an enrichment score. We aggregated the median ranks (normalized to total number of expressed probes) of the four voxels with the highest median ranks. We subtracted the aggregated normalized median ranks from 1 and used the result as the final score for each region, so that a score close to 1 indicates an enrichment of the cluster marker genes in the top ranked probes of the voxels of each region. In Supplementary Table 8, we have reported the top ten results for each cluster.

To build one final regional prediction, we assigned the highest scoring region as the possible spatial origin (if the score was >0.8). However, to further refine the prediction, we included the known dataset origin for many studies. For each subregion-specific dataset, we manually curated a set of likely Allen Brain Atlas regions and checked their contribution to the cells of each cluster. On the basis of this, we down-scaled the median rank predictions if insufficient cells originated from the predicted region (for example, a high mammillary region score was down-scaled if the majority of cells originated from preoptic/anterior datasets). We further checked many clusters manually and changed the final annotation shown in the paper figures to the second- or third-highest prediction for well-documented cell types. To reduce the number of categories for the color-coded annotation in Figure 2a, we summarized some regions by spatial proximity (for example, grouping all mammillary and pre-mammillary clusters or all preoptic region clusters together).

### Mapping of new data

We stored the reference scVI model using scvi.model.SCVI.save. The scvi.model.SCVI.load_query_data method allows the embedding of a query dataset into the latent space of the reference map via scArches algorithm^15,63^. We used Seurat’s ProjectUMAP function to calculate nearest neighbors in the reference and visually embed a query dataset into the original UMAP^9^. The nearest neighbors *N*_*c*_ were used to propagate cluster labels by calculating the mapping probability per available label *y* using the similarity to each neighbor: $p\left( {Y = y|X = c} \right) = \frac{{\mathop {\sum}\limits_{i{\it{\epsilon }}{{{\mathrm{N}}}}_{\it{c}}} {I\left( {y^{\left( i \right)} = y} \right) \times sim(c,n_i)} }}{{\mathop {\sum}\limits_{j{\it{\epsilon }}{{{\mathrm{N}}}}_c} {sim(c,n_j)} }}$ where similarity to each neighbor *I* is the indicator function and *y*^(*i*)^ is the label of *i*th neighbor. This approach is adapted from the scArches algorithm^63^, but simplified by skipping the gaussian smoothing step and using cosine similarity derived from the cosine distances as: $sim_{cosine} = 1 - \frac{{dist_{\rm{cosine}}^2}}{2}$. We combined the mapping and projection functions together with preprocessing and visualization functions in an R package: mapscvi (https://github.com/lsteuernagel/mapscvi). In order to map data from Romanov et al.^20^, we used the functions predict_query and project_query from the mapscvi package to construct a projected embedding and clustering of the data. For nearest-neighbor detection, we used *k* = 25 neighbors.

We employed HypoMap to explore bulk bacTRAP data of *Agrp*, *Pomc*, *Pnoc*, and *Glp1r* neurons. Comparison of the reference samples (’input’) with the immunoprecipitation (’IP’) with DEseq2 (ref. ^64^) yielded signatures of genes that are enriched in the IP and likely to be expressed in the targeted cell types. We adapted rank-biased overlap (RBO)^54^ to compare two sets of ranked genes: (1) bacTRAP signature of interest ranked by the their log_2_(fold change); and (2) cluster markers of each cluster in the sc-seq reference ranked by their log_2_(fold change) or specificity scores. We used extrapolated RBO for uneven lists as implemented in the gespeR R package (version 1.26.0) to calculate RBO scores for each bacTRAP signature on each cluster of the mrtree clustering^54^ (https://www.bioconductor.org/packages/release/bioc/html/gespeR.html).

### NucSeq comparative analysis

To compare the molecular profiles of single-cell and single-nucleus data in HypoMap, we calculated the correlation of gene expression between our in-house nucSeq and the public sc-seq datasets. For the comparison, we used the projected level 5 clustering (C185, corresponding to the lowest level of the hierarchical tree in Figs. 2a and 4c). We selected genes that were expressed in more than 20% of cells of a cluster or that had a mean expression > 0.2 (log-normalized) in at least one cluster. Using the mean expression per cluster (single-cell versus nucSeq), we calculated Pearson’s *r* for each gene and grouped genes by different classes on the basis of the Ingenuity pathway analysis (Qiagen) for further analysis. For neuropeptides we included genes annotated with the gene ontology (GO) term GO:0005179 or annotated ko04080 in the KEGG pathway. In order to compare the correlation of sc-seq and nucSeq on the cluster level, we used marker genes of each cluster with specificity > 0.25 and Pearson’s *r* > 0.3. Pearson’s *r* for each cluster was calculated between nucSeq and HypoMap or the individual sc-seq datasets in HypoMap across all marker genes.

We used a curated set of IEGs from Wu et al.^17^ and reduced the set to 73 genes (expressed in >300 cells and a maximum of 10,000 cells in nucSeq). We manually added *1700016P03Rik*, which correlated with *Fos*. To determine whether *1700016P03Rik* is a CREB1-target gene, we looked for cAMP response element (CRE) in the promoter^65^. Briefly, we used BSgenome (version 1.58) (https://bioconductor.org/packages/release/bioc/html/BSgenome.html) to retrieve promoter sequences –800 to +200 base pairs (bp) around the transcription start site (TSS) (mm10). We then used refTSS v3.3 to obtain a set of TSSs per gene^66^. Detection of JASPAR profiles MA0018.1 and MA0018.2 from JASPAR2020 (ref. ^67^) was conducted with TFBStools (version 1.28) (https://bioconductor.org/packages/release/bioc/html/TFBSTools.html) using the function searchSeq on both strands and with a relative score of >80%. We counted the number of detected profiles per promoter and normalized it by the number of non-overlapping promoters per gene. We found multiple occurrences of CRE in *1700016P03Rik*’s promoter, similar to other known CREB1-target genes (Extended Data Fig. 7b). To quantify the activation of clusters (Level 4) we counted the number of significantly up-regulated IEGs detected using the Wilcoxon test implemented in Seurat’s FindMarkers per cluster and split by diet condition, using Bonferroni correction for multiple testing.

Differential expression between cells from fasted and ad-libitum-fed mice was performed using the Wilcoxon test and negative binomial generalized linear model implemented in Seurat’s FindMarkers function (see Supplementary Tables 10 and 11). The enrichment of significantly up-regulated genes in the fasted state in AgRP neurons for GO ‘Biological Process’ terms was conducted with the clusterProfiler R package (version 3.18.1)^68^ (https://bioconductor.org/packages/release/bioc/html/clusterProfiler.html). We used the enrichGO function with default parameters and all genes expressed in at least 10% off all AgRP neurons in nucSeq as background gene set. The simplify function was used to remove redundant terms

### Experimental methods

All experimental methods regarding animal care and mouse lines, details on the bacTRAP, nucSeqm and in situ hybridization experiments can be found in the Supplementary Information.

### Statistics

Statistical analyses are described in the respective sections. No prior power calculations were performed. For large-scale hypothesis testing (for example, marker genes or differential gene expression) dedicated R packages for these analyses were used and included correction for multiple testing (DESeq2: Benjamini–Hochberg, Seurat: Bonferroni). For individual null hypothesis tests, we used the appropriate functions in R: t.test, wilcox.test, or cor.test with alternative set to ‘two.sided.’ For the quantification in Figure 7, we additionally used functions from the R packages ggpubr (version 0.4.0) (https://github.com/kassambara/ggpubr) and rstatix (version 0.7.0) (https://github.com/kassambara/rstatix).

### Reporting summary

Further information on research design is available in the Nature Research Reporting Summary linked to this article.

## Supplementary information

Supplementary Information Supplementary Methods

Reporting Summary

Supplementary Tables 1–21 See data dictionary included in the .xlsx file for a detailed description of tables and columns.

## Data Availability

Both HypoMap and the hypothalamic nucSeq are available in an interactive CellxGene viewer (available via https://www.mrl.ims.cam.ac.uk). Additionally, the Seurat object containing the HypoMap, which is required to reproduce the shown figures and to project new data, is deposited at University of Cambridge’s Apollo Repository (10.17863/CAM.87955) in standard RDS format. The nucSeq and the bacTRAP profiling data for *Agrp*, *Glp1r*, and *Pomc* neurons are available from the Gene Expression Omnibus (GEO), accession numbers: GSE207736 and GSE208355, respectively. The *Pnoc* bacTRAP data are available at GSE137626. The *Pomc*-*Lepr* and *Pomc*-*Glp1r* bacTRAP data are available at GSE153753. The published sc-seq studies used to construct HypoMap are listed in Supplementary Table 1. Source data are provided with this paper.

## Source data

Source Data Fig. 1 Single cell UMAP data
Source Data Fig. 2 Hierarchical tree annotations, edgelist and marker gene information
Source Data Fig. 3 Single cell UMAP data and cluster comparison tables
Source Data Fig. 4 Statistical source data and single cell UMAP data
Source Data Fig. 5 Single cell UMAP data, hierarchical tree annotations, edgelist and correlation data
Source Data Fig. 6 Statistical source data and single cell UMAP data
Source Data Fig. 7 Statistical source data and single cell UMAP data
Source Data Extended Data Fig. 1 Statistical source data
Source Data Extended Data Fig. 2 Statistical source data and single cell UMAP data
Source Data Extended Data Fig. 3 Single cell UMAP data
Source Data Extended Data Fig. 4 Hierarchical tree annotations, edgelist
Source Data Extended Data Fig. 5 Hierarchical tree edgelist
Source Data Extended Data Fig. 6 Single cell expression data
Source Data Extended Data Fig. 7 Statistical source data and single cell UMAP data
Source Data Extended Data Fig. 8 Single cell UMAP data
Source Data Extended Data Fig. 9 Single cell UMAP data

## Code availability

The code used to create all Figures, all additional input data, the output plots, tables and source data files can be found at: https://github.com/lsteuernagel/hypoMap_paper. An R package that allows mapping of new single-cell data onto the existing HypoMap scVI model is available at: https://github.com/lsteuernagel/mapscvi. The pipeline for evaluation of scVI hyperparameters to optimize the HypoMap model can be found at https://github.com/lsteuernagel/scIntegration. The pipeline to build HypoMap, including all downstream clustering and annotation steps, can be found at: https://github.com/lsteuernagel/scHarmonization.

The R packages scUtils (https://github.com/lsteuernagel/scUtils) and scCoco (https://github.com/lsteuernagel/scCoco) provide additional functions used in the above pipelines. We will provide any other code upon request.
