# Supplementary material for: HypoMap—a unified single-cell gene expression atlas of the murine hypothalamus
Source: Nat Metab. 2022 Oct 20;4(10):1402–19. doi: 10.1038/s42255-022-00657-y (PMC9584816; doi:10.1038/s42255-022-00657-y)
Supplement: Supplementary file 1 — Supplementary Methods [file 42255_2022_657_MOESM1_ESM.pdf]

nature metabolism

Article

https://doi.org/10.1038/s42255-022-00657-y

HypoMap—a unified single-cell gene expression atlas of the murine hypothalamus

In the format provided by the authors and unedited

Supplementary Information

Single cell data integration

Evaluation pipeline

Our evaluation pipeline to build the hypothalamus reference map consists of a series of R and python scripts, utilizing Seurat, scanpy (version 1.5.1) and scvi-tools (version 0.10.0) [1 2 3], optimized to be used with slurm as job management system to run memory and time consuming tasks on a high-performance computing infrastructure. We used SeuratDisk (version 4.0.2) (https://github.com/mojaveazure/seurat-disk) to convert between h5Seurat and h5ad files. Starting with the merged dataset, we first built a reference map containing a subset of 85,000 neurons to compare different integration algorithms and pipeline approaches.

Various normalization approaches have been proposed to cope with special properties of sc-seq data such as zero-inflation and have also been compared using benchmark datasets [4 5 6]. In a first exploratory analysis, we compared methods that performed well and are accessible: scran [7], sctransform [8] and library-size normalization plus log-transformation (log-normalization) as implemented in Seurat [1] and scanpy [2]. We did not observe a strong improvement in integration performance over standard log-normalization and consequently used this as input to methods that require normalized data. Selection of highly variable genes is another critical step in the processing pipeline: we used Seurat's *FindIntegrationsFeatures* with 'method' set to 'vst' to find features per batch and then took the median rank to prioritize features that are relevant in multiple batches instead of characterizing individual batches. We then scaled the data and calculated a PCA without further corrections as a reference for 'non-integrated' data and as input to Harmony.

With our evaluation pipeline (Ext. Figure 1A), we tested different combinations of feature set sizes, numbers of principal components and integration algorithms to determine which algorithm would be best suited to integrate the full merged dataset with regard to the metrics described below and which hyperparameters would be optimal.

Evaluation of integration Methods

Preventing integration methods from over-correcting and removing biologically relevant cell types is a fundamental problem of large integration tasks, and it is difficult to control for this on the level of neuronal (sub)types. Previous efforts have focused on merging major cell types, such as oligodendrocytes, neurons or astrocytes, and it is possible to harmonize author annotations at this level and use them to evaluate cell type purity [9 10]. However, in most cases these annotations are not precise enough to ensure that neuronal subtypes, specifically subtypes of distinct genetic

and functionally defined neuron types, for example arcuate POMC neurons, are properly represented in the integrated data. Hence, we curated a set of signatures representing well-described and validated subtypes from the included sc-seq datasets and identified cells enriched for these signatures using AUCell (see methods), independently of other annotations or clustering results, to measure biological purity (Suppl. Table 2, Ext. Figure 2E). The goal of the data integration efforts was then to maximize the co-clustering of the same neuronal subtypes from different datasets (mixing), while at the same time keeping different subtypes well separated (purity).

The integration algorithms Seurat (CCA), Harmony, Scanorama, scVI and combat (see methods for a brief introduction) were evaluated with different parameter combinations and input features to identify an optimal reference embedding of the merged data. While various metrics have been proposed to evaluate integration results [10] [11], we adapted nearest-neighbor and clustering based approaches and additionally developed our own metric to rank the integration results. We focused on four metrics: (1) a random-forest mixing score based on how well a classifier was able to differentiate between batches (rf-mixing), (2) k-nearest neighborhood entropy based on the distribution of batches to evaluate local mixing of cells (knn-mixing)[12], (3) average silhouette width to measure cluster separation (ASW) [10] and (4) a nearest neighborhood score based on curated cell types to measure biological purity (knn-purity).

Integration methods overview

Based on available reviews [10] [12] [13] we selected a subset of 5 methods that are established approaches in the sc-seq integration field, performed well on benchmark datasets, are scalable to large amounts of data and represent different algorithmic concepts. We used them to correct for the Batch-ID variable defined based on dataset of origin and the additional detection procedure described above.

Harmony uses a softclustering-approach that allows cells to be assigned to multiple clusters in combination with a correction step to iteratively correct an input embedding (PCA) [14]. We used the *HarmonyMatrix* function from the Harmony R package (version 0.1) (https://github.com/immunogenomics/harmony) to correct the PCA space at different dimensionalities and further tuned some of the available parameters (Theta, Sigma, Lambda). Harmony relies on an initialization via *k-means* clustering, which we repeated with a higher number of random starts (100-200) to make this step more robust.

Scanorama builds on the identification of nearest neighbors in other batches combined with the concept of *panorama stitching* from image analysis to remove batch effects [15]. We provided either the normalized or the scaled counts of the highly variable features as input to obtain a low-

dimensional embedding for evaluation and clustering. We applied the *integrate* function from the original Scanorama package (version 1.7) (https://github.com/brianhie/scanorama) with varying values of the alpha, sigma and knn parameters. With the *correct* function Scanorama is able to correct the original counts as well.

Seurat-integration's approach of *FindIntegrationAnchors* and *IntegrateData* combines pair-wise computation of a shared low dimensional embedding of two batches using canonical correlation analysis (CCA) and identification of nearest neighbors in the other dataset with subsequent iterative merging of all pair-wise results [16]. Here we applied Seurat's pipeline to the log-normalized data and subsequently calculated a PCA on the integrated counts, without varying parameters of the integration itself due to its long runtime.

Combat is an empirical Bayes framework based batch-correction method developed for microarray data that has been shown to perform well on sc-seq data [10] [17]. We used either the normalized or the scaled counts of the highly variable features as input to scanpy's function of the python implementation of combat [2] (https://github.com/brentp/combat.py) to obtain corrected counts which were used to calculate a low-dimensional embedding via PCA for evaluation and clustering.

scVI. Single-cell variational inference models the counts as samples drawn from a zero-inflated negative binomial (ZINB) distribution accounting for library depth and batch origin, utilizing neural networks to map to a low-dimensional latent space and corrected counts as output [18]. It uses the raw UMI counts as input and includes library size as a model parameter, consequently only the feature selection was dependent on the processing pipeline. We used a standard workflow of scvi (v0.16.4) as described in the documentation (https://scvi-tools.org/), tuning the available parameters (max epochs, n layers, n hidden, n latent, dropout-rate) to train a model that best fitted our combined hypothalamus dataset.

Evaluation metrics

In order to prioritize integration results we established four metrics to evaluate batch mixing and cell type purity, partly using established metrics as discussed for example by Lütge *et al.* or Luecken *et al.* [11] [10], and some approaches developed specifically for HypoMap.

ASW: Average silhouette width has been previously used to control separation of cell types after data integration [10]. We used the scikit-learn implementation (https://scikit-learn.org/stable/modules/generated/sklearn.metrics.silhouette_score.html) to calculate average silhouette width based on cosine distances per cell and then summarized this by taking the mean over all cells. Calculating ASW requires cell type or cluster annotations for each cell. Since no high-resolution annotation for all cells is available, we iteratively ran Leiden clustering on a nearest-neighbor tree

of each integration until a pre-specified number of clusters was reached. These steps were implemented using scanpy and ran on a subsample of all cells (38,000) to limit the run-time. It should be noted that ASW does not control for true cell type purity, if a method wrongly groups unrelated cells in well-defined clusters. Additionally, the clustering underlying the ASW calculation differs between integration results, hence the metric can vary even if true separation is similar. ASW above 0 indicates higher cluster separation.

rf-mixing: The random-forest based mixing metric was inspired by our within-dataset batch identification approach. For each integration result we trained a random forest predicting the batch variable on a subsample of all cells (38,000) to limit the run-time. If the random forest is able to identify the batch of origin, only this batch will have a high out-of-bag (oob)-probability, conversely if the random forest cannot distinguish between batches, the oob-probabilities will be distributed more evenly. We quantified the batch prediction per cell as the entropy of the oob-probabilities $p(X_i)$ normalized by the logarithm of the number of batches b with an oob-probability greather than 0.01: $prediction = \frac{-\sum_{i=1}^{b} p(X_i) * \log(p(X_i))}{\log(\sum_{i}^{b} I(p(X_i) > 0.01))}$ where I is the indicator function. While this normalization factor is only an approximation, it ensured that cells from cell types consisting of only few batches were not scoring artificially worse. We then used the median over all cells as the rf-mixing score, with values close to 1 indicating high mixing and values close to 0 indicating full separation of batches.

knn-mixing: This is an implementation of the metric described in Lütge *et al.* [11] and Luecken *et al.* [10], which is based on the k-nearest neighbor tree of the data. knn-based metrics are the most common approach to quantify batch mixing. The entropy of the batch distribution of each cell's neighbors represents an estimator for the local mixing around each cell. We used Seurat's implementation of nearest neighbor identification with Annoy (https://github.com/satijalab/seurat/) with cosine distances to identify 20 neighbors per cell and then calculate the entropy per cell. The knn-mixing score is calculated as the median of the entropy per cell, with higher values indicating better mixing. Overall, we found that rf-mixing and knn-mixing correlated well, but differed in some details: the knn based approach was better at stratifying closely related results, but suffered from hubness: the k-nearest neighbor graph (knn) computed to determine neighbors of each cell was not balanced, instead some cells occur more often as neighbors than others [19]. These cells influenced the metric much stronger than cells that had only one or few neighbors. Especially in the case of unbalanced dataset sizes or contribution to clusters a method that is able to move small batch preferentially into hub positions of the knn graph will score over-proportionally well. For example, we found that Scanorama performed over-proportionally well on knn-based metrics compared to rf-mixing and visual inspection.

knn-purity: Controlling for biologically relevant cell type purity is another obstacle in evaluating integration results. The heterogeneity of neuron populations and their differing annotations by study authors make it difficult to use these as a common basis in evaluation metrics. We decided to curate a set of known cell type signatures, which characterize ground-truth cell types in the integrated dataset independently of specific clustering results or annotations. We started by constructing signatures from RNA-seq data of genetically defined cell types generated as part of the Neuro-seq dataset [20], but eventually decided that these did not cover a sufficient number of different cell types and lacked specificity. We then turned to building an initial integration of the data with the two easily accessible methods scVI and Harmony using default parameters. Inspection of the resulting UMAPs showed overall similarity of the two, hence we next used Leiden clustering on the scVI result to obtain cluster labels and then defined informative marker genes for a subset of these: clusters with at least 3 distinct marker genes, indicated by specificity scores (see Marker detection) above 3 and a restricted number of occurrences as marker genes across all clusters were selected for further inspection. Next, we manually curated this list of clusters, requiring them to be described in at least one of the original studies and preferably validated through staining or an in-depth description. The signature of specific marker genes of each cluster was mapped back onto the dataset using the AUCell R package (version 1.12.0) [21] to select cells belonging to the respective clusters (Suppl. Table 2). AUCell computes a score based on the placement of the signature genes within the ranked gene expression values of each cell, which is independent of the data integration or clustering. We chose this indirect approach to prevent tailoring our metric towards the clustering used to define the cell types, at the cost of losing accuracy, as not all cells are confidently mapped when choosing a restrictive threshold for the AUC-score per cell. AUCell was used with default parameters and aucMaxRank set to 700 genes. We used the "global" distribution based on mean and standard deviations of all cells as proposed by the package authors (*AUCell exploreThresholds*) scaled by a user-defined factor of 2.5 as threshold to define which cells belong to a cell type [21]. We additionally used the unified author annotations of major cell types (e.g., astrocytes, oligodendrocytes) to define signatures in a similar way and used them together with the neuron cell types during the mapping with AUCell. In total we defined 24 neuron types and 10 non-neuronal cell types (Suppl. Table 2) that mapped to 145,984 cells (37.9% of all cells) in HypoMap (Ext. Figure 2E). The knn graph defined for knn-mixing was used to calculate the fraction of cells in the neighborhood that belong to the same cell type for each cell of a cell type. For each cell type we averaged the value over all mapped cells to obtain a per-cell type knn-purity score and then used the global median over these values to rank the methods.

For visualization and final ranking we normalized each metric to a range of 0 to 1 between the minimum and maximum scores of all results. For knn-purity we set the minimum to 0.25 to better

reflect that all results retained some purity. The purity score as depicted in Figure 1a was then calculated as 75% of the knn-purity score plus 25% of ASW (or only using knn-purity if ASW was shown separately such as in Ext. Figure 2) and the global mixing score as 50% of knn-mixing and 50% of rf-mixing.

Comparison of integration methods for HypoMap

For our initial evaluation of integration methods we used 12 datasets (Suppl. Table 1) and subset them to neuronal cell types as they provided the biggest challenge during the integration of the sc-seq data. For example, while initial testing showed that all integration methods were able to successfully segregate major cell types such Astrocytes and Oligodendrocytes, separation of complex cell types such as the *Qrfp*-expressing neurons from hypocretine (*Hcrt*)-expressing neurons was not possible with all methods. For the systematic comparison of all five integration methods we used a heterogeneous dataset of 85,451 cells to evaluate more than 1,000 combinations of selection approaches and highly-variable feature set sizes, embedding sizes and integration method parameters with the four metrics described above (Ext. Figure 1). For the visualization and global ranking, we computed a final mixing score as the average of the rf-mixing and the knn-mixing scores summarized over all cells. We defined a global purity score as 75% of the knn-purity score plus 25% of ASW, giving less weight to ASW because it can also score highly if clusters are not biologically meaningful but well separated.

As shown in Ext. Figure 1B, both scVI and PCA (without further integration, Raw) showed the best retention of the cluster purity, but scVI consistently achieved much higher dataset mixing scores. Harmony and Seurat integration also performed well in dataset mixing, but had lower cluster purity, consistent with previous findings[9] [10]. Scanorama was previously reported to perform well[10] and achieved high scores in both purity and separation in our study, but the mixing score remained unsatisfactory. Combat exhibited good mixing scores and showed good retention of known cell types (purity), however, the cluster separation (ASW), was inferior compared to scVI (Ext. Figure 1B, Suppl. Table 3). Hence, for the final HypoMap integration we selected scVI to integrate the data sets.

Optimizing scVI for HypoMap

We used the metrics and cell type signatures described above to determine the best combination of hyperparameters (Ext. Figure 2) based on a random-search like approach where we tested a random subset of a large grid of possible parameter combinations to decrease the total run-time. We also explored whether there was a difference in integration performance when sub-setting to neurons only. For this, we trained scVI with the same hyperparameter sets for the auto-encoder (but different feature sets) on the full data and a subset containing all neurons. The evaluation

was only conducted on the neurons (for both full data and neuronal subset) to avoid scores of non-neuronal cell types influencing the metrics in either direction. We found that sub-setting to neurons had no clear advantage in terms of mixing or purity (Ext. Figure 2b). Therefore, we continued without splitting the data, which also allowed for a more straightforward downstream analysis of the results. To define a robust set of final parameters we compared different hyperparameter ranges across the metrics similar to the example shown in Ext. Figure 2c.

For the final scVI model we found that a relatively high number of training epochs led to better mixing at the cost of cell type purity. The final model was trained for 300 epochs. We increased the number of n layers to 3 and used 256 nodes per layer (n hidden) in the final model, although the difference to 128 nodes was very small. Compared to other methods, scVI profited less from a higher dimensionality of the latent space, but the default values of 10 or 20 for n latent led to a worse purity performance than values for n latent between 50 and 120. For the final model we set n latent to 85. All other parameters were set to default.

Experimental Methods

Animal husbandry

All animal procedures were conducted according with protocols approved by local government authorities (Bezirksregierung Köln). Permission for breeding and experiments on mice was issued by the Department for Environment and Consumer Protection-Veterinary Section in Cologne. Mice were housed in individually ventilated cages at 22–24°C and at 45-55% humidity, using a 12-h light/dark cycle. Animals had access to water and food *ad libitum* and were fed a normal chow diet (ssniff, V1554). Food was only withdrawn during defined fasting periods.

Mouse studies performed in Cambridge were in accordance with UK Home Office Legislation regulated under the Animals (Scientific Procedures) Act 1986 Amendment, Regulations 2012, and procedures were approved by the University of Cambridge Animal Welfare and Ethical Review Body. 18 male C57BL/6J mice at 6-8 weeks were housed in individually ventilated cages with temperature controlled at 20-24°C and humidity at 45-65%, and a 12-h light/dark cycle (lights on 06:00–18:00) at the animal facility at the Anne McLaren Building, University of Cambridge, Animals had *ad libitum* access to food (RM3(E) Expanded chow, Special Diets Services, UK), except for the overnight fasted group (6 animals), where the chow was removed from at 5pm until 9am the next day, all animals had access to water throughout the experiment. Animals were randomized for the grouping.

Mouse lines

<u>*Driver lines*</u>

Glp1r-ires-Cre [22], AgRP-ires-Cre [23] and POMC-Cre [24] mice have been previously described.

ROSA26lSlEGFPL10a (ROSA26-CAGS-lox-STOP-lox-EGFPL10a-WPRE) mice

This line was generated by breeding ROSA26lSlrSrEGFPL10a (ROSA26-CAGS-lox-STOP-lox-roxSTOP-rox-EGFPL10a-WPRE) [25] with a ubiquitously expressed CAGGS-Dre deleter line [26]. *Experimental lines*

C57BL/6N mice were obtained from Charles River, France.

Glp1r^{Cre} ROSA26lSlEGFPL10a mice were generated via mating homozygous Glp1r-ires-Cre mice to homozygous ROSA26$^{fl/fl}$ mice of the EGFPL10a construct. A similar breeding strategy was used for the POMC^{Cre} ROSA26lSlEGFPL10a mice. Resulting double transgenic Cre$^{+/-}$ ROSA26$^{fl/wt}$ mice were used as experimental animals.

Sex and gender differences

For the construction of HypoMap we did not have control over the samples of public data sets. In the final HypoMap 48.6% of cells originated from male mice, 24.9% from female mice and for 26.5 % it was not clear due to missing information or pooling of samples. For validation experiments we used male mice, a decision that was mostly driven by the high experimental cost of nucSeq and bacTRAP and by the observation that most HypoMap clusters are containing both female and male cells and thus observations about general gene expression (like the ISH experiments) should be transferable between males and females.

BacTRAP-based ribosomal profiling

Affinity purification of translating ribosomes was performed as described by Heiman *et al.* [27] with minor modifications. Briefly, 10 weeks old male Glp1r^{Cre} ROSA26lSlEGFPL10a mice, 12 weeks old POMC^{Cre} ROSA26lSlEGFPL10a and 12 weeks old AGRP^{Cre} ROSA26lSlEGFPL10a were sacrificed in a random-fed state. The hypothalamus was rapidly dissected and immediately snap frozen in liquid nitrogen until use. Please see Suppl. Table 21 for details on all reagents and chemicals used in the following sections.

Protein A Dynabeads (375µl per IP; Invitrogen) were prewashed and 50µg of two anti-GFP antibodies (HtzGFP-19F7 and Htz-GFP-19C8, Memorial Sloan Kettering Monoclonal Antibody Facility) were added to the beads and incubated at 4°C overnight. Then, the bound Protein A Dynabeads were washed three times and resuspended in 200µl wash buffer.

Pooled hypothalamic tissue (4 mice pooled for each Glp1r^{Cre} ROSA26lSlEGFPL10a replicate, 4 replicates; 3 mice pooled for each POMC^{Cre} ROSA26lSlEGFPL10a replicate, 4 replicates; 3 mice pooled for each AGRP^{Cre} ROSA26lSlEGFPL10a replicate, 3 replicates) was homogenized in lysis

buffer on a rotating glass/teflon potter homogenizer (Potter S, Braun) at 4°C. Homogenates were centrifuged at 2,000x*g* for 10 min at 4°C, and the supernatant was transferred to a new microcentrifuge tube on ice and NP-40 (Applichem) and 1,2-diheptanoyl-sn-glycero3-phosphocholine (DHPC; Avanti Polar Lipids) were added to the supernatant at a final concentration of 1% and 30 mM, respectively. After incubation on ice for 5 min, the lysate was centrifuged at 17,000x*g* for 10 min at 4°C and 30µl of supernatant was snap frozen in liquid nitrogen until use.

For immunoprecipitation, 200µl of anti-GFP antibody-bound Protein A magnetic beads were added to the supernatant and incubated at 4°C for 1h. Next, beads were collected with a magnet and subsequently washed four times. After the final wash the beads were collected. Input and IP beads were resuspended and incubated in RLT buffer (RNeasy Micro Kit, QIAGEN) for 5 mins at room temperature (RT). RNA was purified subsequently using the RNeasy Micro Kit (QIAGEN). RNA integrity was assessed using an Agilent 2100 bioanalyzer.

RNA Sequencing

Whole transcriptome amplification was performed using the Ovation RNA-seq system (V2) (Tecan, Mannedorf, Switzerland). Sequencing libraries were generated using Illumina Nextera XT DNA sample preparation kit (San Diego, CA, USA.) using 1 ng cDNA input, and were paired-end sequenced (2x 100bp) on an Illumina HiSeq 4000.

Analysis of bacTRAP RNA-sequencing data

We applied the community-curated nfcore rnaseq analysis pipeline (version 1.4) (https://nf-co.re/ rnaseq). The gene-level quantification was carried out using Salmon (version 0.14.1) [28] using the reference genome GRCm38. In order to obtain characteristic signatures representing the molecular profiles of targeted neurons we identified up-regulated genes ($pvalue_{adjusted} \leq 0.01$, $log_2(FoldChange) > 0.5$) between the ribosomal pulldown (IP) and the hypothalamic background (Input) using differential gene expression analysis with the DESeq2 R package [29] (version 1.30.0). We additionally filtered for protein-coding genes using Ensembl [30]. For the Pomc-Lepr, Pomc-Glp1r and *Pnoc* bacTRAP data we used published results [25 31].

Single-nucleus RNA Sequencing

Single-nucleus RNA sequencing was performed as previously described [32]. Briefly, hypothalami were pooled by the nutritional condition to yield 2 *ad libitum* fed samples (prepared on separate days) and 1 fasted sample. The samples were homogenized using a Dounce homogeniser in homogenate buffer containing 1 µl/ml of DRAQ5 (Biostatus, Loughborough, UK). The homogenates were centrifuged at 900×*g* for 10 min at 4 °C, the pellets were resuspended in 25%

OptiPrep (Sigma Aldrich) solution, and was layered on top of separate 29% OptiPrep solutions to create a density gradient for nuclear separation at 13,500×*g* for 20 min at 4 °C. The nuclear pellet was removed and resuspended in a wash buffer and was passed through a 40 μm cell strainer for Fluorescent-activated cell sorting (FACS) on a BD Influx cell sorter (BD Biosciences, Franklin Lakes, NJ, USA). The gating was set according to FSC and SSC to and fluorescence at 647/670 nm to detect DraQ5 nuclear staining. Each sample was sorted into two separate tubes, each with 15,000 particles.

Sequencing libraries were generated using 10X Genomics Chromium Single-Cell 3′ Reagent kits (Pleasanton, CA, USA, version 3) and cDNA was PCR amplified for 19 cycles. Paired-end sequencing was performed using an Illumina NovaSeq 6000 (San Diego, CA, USA, read 1: 28 bp and read 2: 91 bp).

RNA *in situ* hybridization

After a 16 h fast, 10 weeks old male C57BL/6N mice were perfused transcardially with ice-cold PBS (pH 7.4) followed by ice-cold 4% paraformaldehyde (PFA; in PBS, pH 7.4). The brain was removed from the skull and post-fixed in 4% PFA at 4˚C for 24 h, and moved to 20% sucrose solution (in 1X PBS) at 4˚C. The brains were cut in 20μm thick sections. The fluorescence ISH (RNAscope) was performed using probes specific for *Pomc* (Cat# 314081), *Anxa2* (Cat# 501011), *Ghrh* (Cat# 470991), *Oxt* (Cat# 493171), *Sst* (Cat# 404631), *Unc13c* (Cat# 519021), *Tbx19* (Cat# 484741), *Nkx2-4* (Cat# 1070491), *Trh* (Cat# 436811), *Glp1r* (Cat# 418851), , *Crabp1* (Cat# 474711), *Tmem215* (Cat# 409241), *Htr3b* (Cat# 497541), *and Nts* (Cat# 420441) according to the manufacturer's instructions. Probes specific for *Pnoc* was custom made and contains 20 oligonucleotide pairs targeting region 325 - 1263 of transcript accession NM_010932.2. 3-plex negative and 3-plex positive control probes (ACDBio) were processed in parallel with target probes. All incubation steps were performed at 40˚C using the ACD HybEz hybridization system (ACDBio). Sections were mounted on SuperFrost Plus (ThermoFisher), dried at RT and baked at 60˚C overnight. Sections were subsequently incubated with hydrogen peroxide at RT for 10 min and rinsed in autoclaved Millipore water two times and subsequently submerged in Target Retrieval Buffer (ACDBio) at 95-97˚C for 8 min. Slides were washed in autoclaved Millipore water for 15 sec and were dehydrated in 100% ethanol for 30 sec. After air dry, a hydrophobic barrier was using ImmEdge barrier pen (ACDBio). Slides were then stored at RT until assaying. Sections were incubated with Protease III (ACDBio) for 25 min. The subsequent hybridization, amplification and detection steps were performed according to the manufacturer's instructions (Multiplex Fluorescent Detection kit v2, ACDBio). Sections were mounted with DAPI with Vectashield Antifade Mounting Medium (Vector Laboratories) and stored at 4˚C in the dark.

Imaging and quantification of RNA ISH

Images were captured as previously described [25]. Briefly, sections were imaged using Leica TCS SP-8-X confocal microscope with a 40X objective, with z-stacks set at 1μm intervals. Images were analyzed using FIJI (National Institutes of Health, version 2.0.0-rc-41/1.50d). For representative images, adjustments were made to brightness and contrast, whereas for quantification, all channels were kept unchanged. Two to four sections per mouse were quantified per area. Cell counting was performed manually. The threshold for probe recognition was determined by manual visual judgment, by defining single-cell regions of interest (ROIs) showing *Pomc*, *Ghrh*, *Oxt*, *Sst*, *Tbx19* or *Trh*, and only four or more probe signals per ROI of *Glp1r*, *Anxa2*, *Unc13c* or *Nkx2-4* were considered as positive. For the Pnoc cell type validation, ROIs were determined in a similar way.

References

1. Hao, Y. *et al.* Integrated analysis of multimodal single-cell data. *Cell* (2021) doi:10.1016/j.cell.2021.04.048.

2. Wolf, F. A., Angerer, P. & Theis, F. J. {SCANPY}: large-scale single-cell gene expression data analysis. *Genome Biol.* **19**, (2018).

3. Gayoso, A. *et al.* scvi-tools: a library for deep probabilistic analysis of single-cell omics data. (2021) doi:10.1101/2021.04.28.441833.

4. Lytal, N., Ran, D. & An, L. Normalization Methods on Single-Cell {RNA}-seq Data: An Empirical Survey. *Front. Genet.* **11**, (2020).

5. Cole, M. B. *et al.* Performance Assessment and Selection of Normalization Procedures for Single-Cell {RNA}-Seq. *Cell Syst.* **8**, 315--328.e8 (2019).

6. Tian, L. *et al.* Benchmarking single cell {RNA}-sequencing analysis pipelines using mixture control experiments. *Nat. Methods* **16**, 479–487 (2019).

7. Lun, A. T. L., Bach, K. & Marioni, J. C. Pooling across cells to normalize single-cell {RNA} sequencing data with many zero counts. *Genome Biol.* **17**, (2016).

8. Hafemeister, C. & Satija, R. Normalization and variance stabilization of single-cell {RNA}-seq data using regularized negative binomial regression. *Genome Biol.* **20**, (2019).

9. Swamy, V. S., Fufa, T. D., Hufnagel, R. B. & McGaughey, D. M. Building the mega single-cell transcriptome ocular meta-atlas. *Gigascience* **10**, (2021).

10. Luecken, M. D. *et al.* Benchmarking atlas-level data integration in single-cell genomics. *Nat. Methods* **19**, 41–50 (2022).

11. Lütge, A. *et al.* {CellMixS}: quantifying and visualizing batch effects in single-cell {RNA}-seq data. *Life Sci. Alliance* **4**, e202001004 (2021).

12. Chazarra-Gil, R., van Dongen, S., Kiselev, V. Y. & Hemberg, M. Flexible comparison of batch correction methods for single-cell RNA-seq using BatchBench. *Nucleic Acids Res.* (2021) doi:10.1093/nar/gkab004.

13. Tran, H. T. N. *et al.* A benchmark of batch-effect correction methods for single-cell {RNA} sequencing data. *Genome Biol.* **21**, (2020).

14. Korsunsky, I. *et al.* Fast, sensitive and accurate integration of single-cell data with Harmony. *Nat. Methods* **16**, 1289–1296 (2019).

15. Hie, B., Bryson, B. & Berger, B. Efficient integration of heterogeneous single-cell transcriptomes using Scanorama. *Nat. Biotechnol.* **37**, 685–691 (2019).

16. Stuart, T. *et al.* Comprehensive Integration of Single-Cell Data. *Cell* **177**, 1888--1902.e21 (2019).

17. Johnson, W. E., Li, C. & Rabinovic, A. Adjusting batch effects in microarray expression data using empirical Bayes methods. *Biostatistics* **8**, 118–127 (2006).

18. Lopez, R., Regier, J., Cole, M. B., Jordan, M. I. & Yosef, N. Deep generative modeling for single-cell transcriptomics. *Nat. Methods* **15**, 1053–1058 (2018).

19. Amblard, E., Bac, J., Chervov, A., Soumelis, V. & Zinovyev, A. Hubness reduction improves clustering and trajectory inference in single-cell transcriptomic data. (2021) doi:10.1101/2021.03.18.435808.

20. Sugino, K. *et al.* Mapping the transcriptional diversity of genetically and anatomically defined cell populations in the mouse brain. *Elife* **8**, (2019).

21. Aibar, S. *et al.* {SCENIC}: single-cell regulatory network inference and clustering. *Nat. Methods* **14**, 1083–1086 (2017).

22. Williams, E. *et al.* Sensory Neurons that Detect Stretch and Nutrients in the Digestive System. *Cell* **166**, 209–221 (2016).

23. Tong, Q., Ye, C.-P., Jones, J. E., Elmquist, J. K. & Lowell, B. B. Synaptic release of {GABA} by {AgRP} neurons is required for normal regulation of energy balance. *Nat. Neurosci.* **11**, 998–1000 (2008).

24. Balthasar, N. *et al.* Leptin Receptor Signaling in {POMC} Neurons Is Required for Normal Body Weight Homeostasis. *Neuron* **42**, 983–991 (2004).

25. Biglari, N. *et al.* Functionally distinct {POMC}-expressing neuron subpopulations in hypothalamus revealed by intersectional targeting. *Nat. Neurosci.* (2021) doi:10.1038/s41593-021-00854-0.

26. Anastassiadis, K. *et al.* Dre recombinase, like Cre, is a highly efficient site-specific recombinase in E. coli, mammalian cells and mice. *Dis. Model. {\&} Mech.* **2**, 508–515 (2009).

27. Heiman, M. *et al.* A Translational Profiling Approach for the Molecular Characterization of {CNS} Cell Types. *Cell* **135**, 738–748 (2008).

28. Patro, R., Duggal, G., Love, M. I., Irizarry, R. A. & Kingsford, C. Salmon provides fast and bias-aware quantification of transcript expression. *Nat. Methods* **14**, 417–419 (2017).

29. Love, M. I., Huber, W. & Anders, S. Moderated estimation of fold change and dispersion for {RNA}-seq data with {DESeq}2. *Genome Biol.* **15**, (2014).

30. Yates, A. D. *et al.* Ensembl 2020. *Nucleic Acids Res.* (2019) doi:10.1093/nar/gkz966.

31. Jais, A. *et al.* {PNOCARC} Neurons Promote Hyperphagia and Obesity upon High-Fat-Diet Feeding. *Neuron* **106**, 1009--1025.e10 (2020).

32. Dowsett, G. K. C. *et al.* A survey of the mouse hindbrain in the fed and fasted states using single-nucleus {RNA} sequencing. *Mol. Metab.* **53**, 101240 (2021).
