# Supplementary material for: HypoMap—a unified single-cell gene expression atlas of the murine hypothalamus
Source: Nat Metab. 2022 Oct 20;4(10):1402–19. doi: 10.1038/s42255-022-00657-y (PMC9584816; doi:10.1038/s42255-022-00657-y)
Supplement: Supplementary file 2 — Reporting Summary [file 42255_2022_657_MOESM2_ESM.pdf]

nature portfolio

Corresponding author(s): Jens C. Brüning

Last updated by author(s): Sep 2, 2022

Reporting Summary

Nature Portfolio wishes to improve the reproducibility of the work that we publish. This form provides structure for consistency and transparency in reporting. For further information on Nature Portfolio policies, see our Editorial Policies and the Editorial Policy Checklist.

Statistics

For all statistical analyses, confirm that the following items are present in the figure legend, table legend, main text, or Methods section.

n/a	Confirmed	
☐	☒	The exact sample size (n) for each experimental group/condition, given as a discrete number and unit of measurement
☐	☒	A statement on whether measurements were taken from distinct samples or whether the same sample was measured repeatedly
☐	☒	The statistical test(s) used AND whether they are one- or two-sided<br>*Only common tests should be described solely by name; describe more complex techniques in the Methods section.*
☐	☒	A description of all covariates tested
☐	☒	A description of any assumptions or corrections, such as tests of normality and adjustment for multiple comparisons
☐	☒	A full description of the statistical parameters including central tendency (e.g. means) or other basic estimates (e.g. regression coefficient) AND variation (e.g. standard deviation) or associated estimates of uncertainty (e.g. confidence intervals)
☐	☒	For null hypothesis testing, the test statistic (e.g. F, t, r) with confidence intervals, effect sizes, degrees of freedom and P value noted<br>*Give P values as exact values whenever suitable.*
☒	☐	For Bayesian analysis, information on the choice of priors and Markov chain Monte Carlo settings
☐	☒	For hierarchical and complex designs, identification of the appropriate level for tests and full reporting of outcomes
☐	☒	Estimates of effect sizes (e.g. Cohen's d, Pearson's r), indicating how they were calculated

Our web collection on statistics for biologists contains articles on many of the points above.

Software and code

Policy information about availability of computer code

Data collection

Image acquisition software: Leica ASX v.3.5.5.19976

Data analysis

We have extensively described our computational pipeline in the methods. Please also refer to the github repositories described in the code availability statement :
The code used to create all Figures, all additional input data and the output plots and tables shown here can be found at: https://github.com/lsteuernagel/hypoMap_paper . An R package that allows quick mapping of new single cell data onto the existing HypoMap scVI model is available at: https://github.com/lsteuernagel/mapscvi . We will share these repositories and any other code upon request, and change the repositories to public access upon publication.

This docker image combines most packages required for working with our scvi and R seurat pipeline: https://hub.docker.com/r/lsteuernagel/r_scvi

Additionally we provide a list of the most relevant software and packages used in this study:
R (version 4.1.0)
Seurat R package (version 4.1.0)
python (version 3.8)
scanpy (version 1.5.1)
scvi-tools (version 0.16.4)
nfcore rnaseq analysis pipeline (version 1.4)
Salmon (version 0.14.1)
DESeq2 R package 63 (version 1.30.0)

10X Genomics Cellranger 6.0.1 and 5.0.1
STAR 2.7.5
Scater R package (version 1.20.1)
scDblFinder (1.6.0)
https://github.com/chris-mcginnis-ucsf/DoubletFinder (V2.0)
randomForest R package (version 4.6)
Scanorama package (version 1.7)
Harmony R package (version 0.1)
AUCell R package (version 1.12.0)
leiden R package (version 0.3.7)
cocoframer R package (version 0.1.1)
mrtree (https://github.com/pengminshi/MRtree) (Version: 0.0.0.9000)
ggtree R package (version 2.4.2)
https://github.com/KChen-lab/stratified-tests-for-seurat
gespeR R package (Bioconductor 0.99.5)
networkD3 R package (version 0.4)
BSgenome (version 1.58)
TFBStools (version 1.28)
rstatix (version 0.7.0)
ggpubr (version 0.4.0)
Fiji (version 2.0.0-rc-41/.50d)
docker (version 1.41)
singularity (version 3.4.1-1)
slurm (version 16.05.6)

For manuscripts utilizing custom algorithms or software that are central to the research but not yet described in published literature, software must be made available to editors and reviewers. We strongly encourage code deposition in a community repository (e.g. GitHub). See the Nature Portfolio guidelines for submitting code & software for further information.

Data

Policy information about availability of data

All manuscripts must include a data availability statement. This statement should provide the following information, where applicable:

- Accession codes, unique identifiers, or web links for publicly available datasets
- A description of any restrictions on data availability
- For clinical datasets or third party data, please ensure that the statement adheres to our policy

Both HypoMap and the hypothalamic nucSeq is made available in an interactive cellxgene viewer (available via https://www.mrl.ims.medschl.cam.ac.uk). Additionally, the Seurat object containing the HypoMap, which is required to reproduce the shown figures and to project new data, is deposited at University of Cambridge's Apollo Repository (doi:10.17863/CAM.87955) in standard RDS format.

The nucSeq and the bacTRAP profiling data of Agrp, Glp1r, and Pomc neurons are available from the Gene Expression Omnibus (GEO), accession numbers: GSE207736, and GSE208355 respectively. The Pnoc bacTRAP data are available from GSE137626. The Pomc-Lepr and Pomc-Glp1r bacTRAP data are available from GSE153753. The published sc-seq studies used to construct HypoMap are listed in Suppl. Table 1).

Field-specific reporting

Please select the one below that is the best fit for your research. If you are not sure, read the appropriate sections before making your selection.

☒ Life sciences ☐ Behavioural & social sciences ☐ Ecological, evolutionary & environmental sciences

For a reference copy of the document with all sections, see nature.com/documents/nr-reporting-summary-flat.pdf

Life sciences study design

All studies must disclose on these points even when the disclosure is negative.

Sample size	No prior power calculations for sample size were performed. Sample sizes (n=3-5) for bacTRAP, nucSeq and RNAscope experiments were based on previous experiments (Biglari, 2021; Jais, 2020). For RNAscope validation we aimed for 4 mice with 2-4 sections (rostral and caudal) analyzed per animal and celltype.
Data exclusions	We removed single cells from the nucSeq that were of low quality (e.g. Doublets or low UMI numbers). For the creation of HypoMap removed cells that were of low quality and additionally removed samples that had fewer than 100 cells left after cell filtering. We did not remove any other data.
Replication	A substantial part of the analysis conducted in this study revolves around replicability and comparability of the different data types. We generally find that the nucSeq data is well comparable with the single-cell data but dependent on covariates such as single-cell technology. The bacTRAP RNAseq data partly recapitulates the findings from the single-cell data but does not exactly replicate the expected cell types.

	Using RNAscope we validated the expression of Glp1r in the cell types that were predicted from the sequencing data.
Randomization	Animals were randomized for the grouping into fed and fasting. For other experiments no experimental condition was tested.
Blinding	Data collection was carried out blinded where applicable. Data analysis of sequencing data (e.g., single-cell data processing and differential expression analysis) was not carried out in a blinded fashion, due to the common workflows requiring availability of metadata and working fully blinded impeding work with these.

Reporting for specific materials, systems and methods

We require information from authors about some types of materials, experimental systems and methods used in many studies. Here, indicate whether each material, system or method listed is relevant to your study. If you are not sure if a list item applies to your research, read the appropriate section before selecting a response.

Materials & experimental systems

n/a	Involved in the study
☐	☒ Antibodies
☒	☐ Eukaryotic cell lines
☒	☐ Palaeontology and archaeology
☐	☒ Animals and other organisms
☒	☐ Human research participants
☒	☐ Clinical data
☒	☐ Dual use research of concern

Methods

n/a	Involved in the study
☒	☐ ChIP-seq
☒	☐ Flow cytometry
☒	☐ MRI-based neuroimaging

Antibodies

Antibodies used	Heintz Lab TRAP anti-GFP 19F7 antibody (Cat# Htz-GFP-19F7, RRID:AB_2716736)<br>Heintz Lab TRAP anti-GFP 19C8 antibody (Cat# Htz-GFP-19C8, RRID:AB_271673)
Validation	From: Heintz Lab; Rockefeller University. Validated in Heiman et al., 2008, DOI:https://doi.org/10.1016/j.cell.2008.10.028<br>Ordered from: Memorial Sloan-Kettering Cancer Center

Animals and other organisms

Policy information about studies involving animals; ARRIVE guidelines recommended for reporting animal research

Laboratory animals	Cologne (bacTRAP & RNAscope):<br>Mice were housed in individually ventilated cages at 22–24◦C and humidity at 45-55% using a 12-h light/dark cycle. Animals had access to water and food ad libitum and were fed a normal chow diet (ssniff, V1554). Food was only withdrawn during defined fasting periods. C57BL/6N mice were obtained from Charles River, France. For RNAscope experiments 16 h fast, 10 weeks old male C57BL/6N mice were sacrificed. For bacTRAP experiments 10 weeks old male Glp1rCre ROSA26lSlEGFPL10a mice, 12 weeks POMCCre ROSA26lSlEGFPL10a and 12 weeks old AGRPCre ROSA26lSlEGFPL10a were sacrificed in a random-fed state.<br>Details on genetically modified mouse lines:<br>Driver lines: Glp1r-ires-Cre (Williams, 2016), AgRP-ires-Cre (Balthasar, 2004) and POMC-Cre (Anastassiadis, 2009) mice have been previously described.<br>ROSA26lSlEGFPL10a (ROSA26-CAGS-lox-STOP-lox-EGFPL10a-WPRE): This line was generated by breeding ROSA26lSlrSrEGFPL10a (ROSA26-CAGS-lox-STOP-lox-roxSTOP-rox-EGFPL10a-WPRE) 7 with a ubiquitously expressed CAGGS-Dre deleter line (Anastassiadis, 2009).<br>Experimental lines: Glp1rCre ROSA26lSlEGFPL10a mice were generated via mating homozygous Glp1r-ires-Cre mice to homozygous ROSA26fl/fl mice of the EGFPL10a construct. A similar breeding strategy was used for the POMCCre ROSA26lSlEGFPL10a mice. Resulting double transgenic Cre+/- ROSA26fl/wt mice were used as experimental animals.<br><br>Cambridge (single nucleus sequencing): Male C57BL/6J mice at 6-8 weeks were housed in ventilated cages in a controlled temperature (20-24°C) and humidity (45-65%) facilities with a 12-h light/dark cycle (lights on 06:00–18:00) and had ad libitum access to food (RM3(E) Expanded chow, Special Diets Services, UK) and water in the animal facility at the Anne McLaren Building, University of Cambridge. For the overnight fasted group (6 animals), the chow was removed from at 5pm until 9am the next day, the animals had access to water throughout the fasted period.
Wild animals	The study did not involve wild animals.
Field-collected samples	The study did not involve samples collected from field.

Ethics oversight	All animal procedures were conducted in compliance with protocols approved by local government authorities (Bezirksregierung Köln). Permission for breeding and experiments on mice was issued by the Department for Environment and Consumer Protection-Veterinary Section in Cologne.<br>Mouse studies performed in Cambridge were in accordance with UK Home Office Legislation regulated under the Animals (Scientific Procedures) Act 1986 Amendment, Regulations 2012, following ethical review by the University of Cambridge Animal Welfare and Ethical Review Body (AWERB)

Note that full information on the approval of the study protocol must also be provided in the manuscript.
